# Oscillation of Cdc20–APC/C–mediated CAMDI stability is critical for cortical neuron migration

**DOI:** 10.1016/j.jbc.2021.100986

**Published:** 2021-07-21

**Authors:** Shohei Okuda, Mariko Sato, Saho Kato, Shun Nagashima, Ryoko Inatome, Shigeru Yanagi, Toshifumi Fukuda

**Affiliations:** 1 Laboratory of Molecular Biochemistry, School of Life Sciences, Tokyo University of Pharmacy and Life Sciences, Hachioji, Tokyo, Japan; 2 Department of Life Science, Faculty of Science, Gakushuin University, Toshima-ku, Tokyo, Japan

**Keywords:** neuron, migration, neurodevelopment, autism, centrosome, Cdc20–APC, CAMDI, APC/C, anaphase-promoting complex/cyclosome, CAMDI, coiled-coil protein associated with MRLC IIa and DISC1, CAMDI-sh, shRNA against mouse *Ccdc141* gene that encodes CAMDI, Cdc20, cell division cycle protein 20, D-box, destruction box, E, embryonic day, EGFP, enhanced green fluorescent protein, HDAC6, histone deacetylase 6

## Abstract

Radial migration during cortical development is required for formation of the six-layered structure of the mammalian cortex. Defective migration of neurons is linked to several developmental disorders such as autism and schizophrenia. A unique swollen structure called the dilation is formed in migrating neurons and is required for movement of the centrosome and nucleus. However, the detailed molecular mechanism by which this dilation forms is unclear. We report that CAMDI, a gene whose deletion is associated with psychiatric behavior, is degraded by cell division cycle protein 20 (Cdc20)–anaphase-promoting complex/cyclosome (APC/C) cell-cycle machinery after centrosome migration into the dilation in mouse brain development. We also show that CAMDI is restabilized in the dilation until the centrosome enters the dilation, at which point it is once again immediately destabilized. CAMDI degradation is carried out by binding to Cdc20–APC/C *via* the destruction box degron of CAMDI. CAMDI destruction box mutant overexpression inhibits dilation formation and neuronal cell migration *via* maintaining the stabilized state of CAMDI. These results indicate that CAMDI is a substrate of the Cdc20–APC/C system and that the oscillatory regulation of CAMDI protein correlates with dilation formation for proper cortical migration.

Cortical neuronal migration is required for normal development of the six-layered structure of the mammalian cerebral cortex (1, 2). Abnormal migration is known to be associated with psychiatric disorders such as autism and schizophrenia and bipolar disorder (3, 4, 5, 6). A migrating neuron travels a long distance past the previously born neuronal population present in the lower layer and reaches the precise layer for which it is intended (7, 8). Neuronal migration involves three repeated steps: creation of a unique structure called the dilation in the proximal region of the leading process, entry of the centrosome into the dilation, and movement of the nucleus and cell soma in the direction of migration (8, 9, 10, 11, 12, 13). Recent studies revealed that Rac1 and its interacting protein POSH localizes activated Rac1 to control the cytoplasmic dilation formation (11) and Cdk5 and its substrates, Dcx and P27kip1, regulate cytoplasmic dilation formation (12). In addition, cerebellar granule neurons (14) and tangential migrating interneurons (15, 16) also formed dilations during migration. Although it has been revealed by knockdown and KO experiments that several genes are linked to abnormal dilation formation, the detailed molecular mechanism and significance of repetitive dilation formation during cortical migration remain unclear.

Anaphase-promoting complex/cyclosome (APC/C) is one type of the ubiquitin ligase complex that functions in the G_2_/M phase in mitotic cells and is known as an important regulator of the cell cycle (17, 18). Substrates of APC/C are recognized by adapter proteins such as cell division cycle protein 20 (Cdc20) and Cdh1 *via* conserved motifs, named destruction box (D-box) and KEN-box, and degraded in a proteasome pathway-dependent manner (19, 20). Recent studies have shown that APC/C ubiquitin ligase also functions in postmitotic neurons. Cdh1–APC/C is involved in axonal growth and patterning (21) and Cdc20–APC/C has been reported to function in dendrite morphogenesis (22). In line with these reports, the APC/C complex controls the development of axon and dendrite functions in postmitotic neurons, but whether neuronal migration is regulated by the complex in mammalian brain development is still unknown.

Coiled-coil protein associated with MRLC IIa and DISC1 (CAMDI) was reported as a novel protein interacting with Disrupted in Schizophrenia 1. CAMDI is localized in the centrosome, and experiments involving its knockdown by shRNA using *in utero* electroporation technology revealed the inhibition of cortical radial migration (23) and GnRH neurons (24). In addition, it was reported that CAMDI regulates cortical radial migration by negatively controlling the activity of histone deacetylase 6 (HDAC6) and promotes the maturation of centrosomes (25, 26). Thus, CAMDI KO mice exhibit psychiatric disorder–like phenotypes. A recent study showed that CAMDI regulates AMPAR cell surface expression and learning and memory through interacting with human memory-associated protein KIBRA (27). These results suggest that CAMDI plays an important role in cortical neuronal migration and that its abnormality is linked to psychiatric and learning-deficit disorders. However, the detailed molecular mechanisms at the stage in which CAMDI functions during neuronal migration have remained unknown.

In this article, we report that CAMDI localizes at both the centrosome and the dilation. CAMDI is stabilized until the centrosome enters the dilation and destabilized immediately thereafter by binding to Cdc20–APC/C. Experiments of CAMDI knockdown and CAMDI D-box mutant overexpression indicated that CAMDI is required for dilation formation and neuronal cell migration. We also describe that oscillation of CAMDI stable/unstable states, which is regulated by Cdc20-APC/C, is critical for cortical neuronal migration, suggesting a novel molecular mechanism in neuronal cell migration.

## Results

### CAMDI is localized at and required for dilation formation of cortical neurons during radial migration

**Figure 1:**
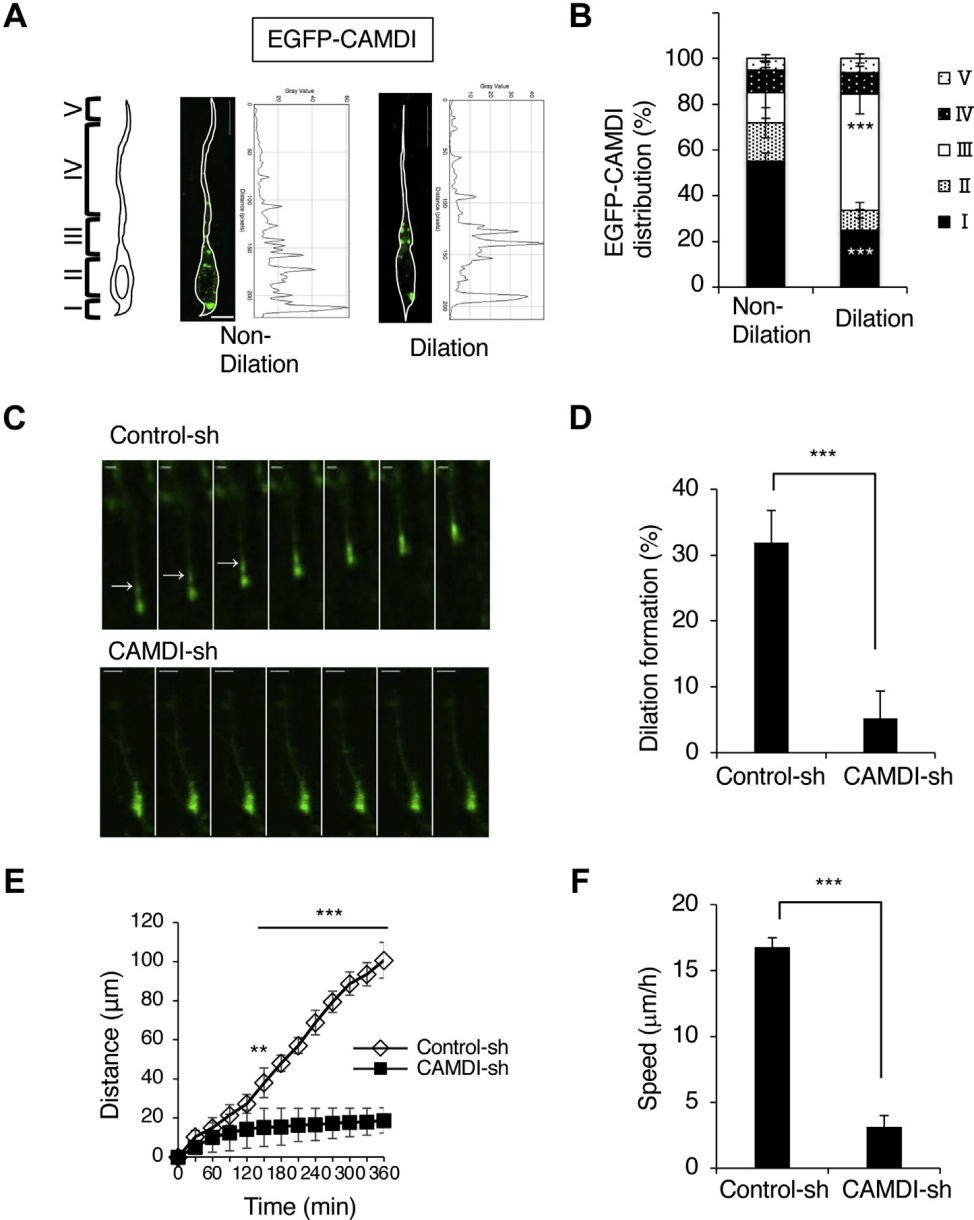
**CAMDI is localized and required for dilation formation of cortical neurons during radial migration.***A*, representative images and line-scan data of single EGFP-CAMDI plasmid electroporated neurons. Coronal sections through the somatosensory cortex of E17.5 were analyzed after *in utero* electroporation at E14.5. EGFP-CAMDI localization was indicated and analyzed by dividing migrating neurons into five zones (I–V). Zone I: the rear of the soma, zone II: soma, zone III: proximal region (<∼20 μm from the front of nuclei) of the leading process, zone VI: leading process, zone V: distal region of the leading process. The scale bar represents 10 μm. *B*, percentage of EGFP-CAMDI fluorescence distribution shown in panel *A*. EGFP-CAMDI is largely localized in zone III in neurons with dilation. n = 3 mice (total 29 cells). ∗∗∗*p* < 0.001, two-way ANOVA followed by Scheffe’s *post hoc* test for differences between dilation and nondilation cells in zones I and III. Data are presented as the mean ± SD. *C*, effect of CAMDI knockdown on dilation formation upon CAMDI-sh expression using time-lapse imaging analysis. The image was taken every 15 min. The scale bar represents 10 μm. *D*, percentage of dilation formation shown in panel *C*. CAMDI knockdown causes inhibition of dilation formation. n = 3 mice/group (control-sh = 99 cells, CAMDI-sh = 116 cells). ∗∗∗*p* < 0.001. Data are presented as the mean ± SD. *E*, migration distance of control-sh or CAMDI-sh electroporated neurons. n = 3 mice/group (control-sh = 10 cells, CAMDI-sh = 10 cells). ∗∗*p* < 0.01; ∗∗∗*p* < 0.001, two-way ANOVA followed by Scheffe’s *post hoc* test. Data are presented as the mean ± SD. *F*, migration speed of control-sh or CAMDI-sh electroporated neurons. n = 3 mice/group (control-sh = 10 cells, CAMDI-sh = 10 cells). ∗∗∗*p* < 0.001, one-way ANOVA with Bonferroni’s *post hoc* test. Data are presented as the mean ± SD. CAMDI, coiled-coil protein associated with MRLC IIa and DISC1; CAMDI-sh, shRNA against mouse *Ccdc141* gene that encodes CAMDI; E14.5, embryonic day 14.5; E17.5, embryonic day 17.5.

In migrating cortical neurons, there is a unique structure, called the dilation, which is formed at the proximal region in the leading process. Although it is known that some proteins regulate the formation of this structure, the detailed molecular mechanism remained unknown. Recent studies showed that CAMDI regulates radial migration during cortical development. Thus, we analyzed whether CAMDI is involved in dilation formation. First, we investigated where CAMDI is localized in migrating neurons forming the dilation. Specifically, we injected enhanced green fluorescent protein (EGFP)-CAMDI plasmid into the mouse embryonic cerebral ventricle by *in utero* electroporation at embryonic day (E)14.5 and subjected the brains to slice culture for analysis at E17.5. After introducing EGFP-CAMDI, we analyzed its intensity in migrating neurons by dividing them into five zones (I–V, Fig. 1*A*) and measured and summed this to calculate the intensity in each zone. The results showed that, while the strongest fluorescence intensity was measured at zone I of the neurons without dilation, in the neurons with dilation, the strongest fluorescence intensity increased at zone III, which is an appropriate region containing the dilation (Fig. 1*B*). To investigate whether CAMDI was required for dilation formation, shRNA against mouse *Ccdc141* gene that encodes CAMDI (CAMDI-sh) with EGFP-expressing plasmid was injected into the mouse embryonic cerebral ventricle and the brains were subjected to live-imaging analysis of electroporated slice culture. Rescue experiment using coexpression of an shRNA-resistant CAMDI plasmid was previously shown in our study (23). In contrast to the normal dilation formation of migrating cortical neurons upon control-sh injection, CAMDI-sh caused a decrease of the dilation formation (Fig. 1*C* and Fig. S1, *A* and *B*). Statistical analysis showed that CAMDI-sh caused severe impairment of dilation formation compared with control-sh (Fig. 1*D*). In a previous study, we performed CAMDI knockdown experiments and created KO mice, which showed impairment of cortical neuronal migration (23, 25). In the present study using live-imaging analysis, we also observed that CAMDI knockdown neurons exhibited impaired radial migration with decreases of migration distance and speed (Fig. 1, *E* and *F*). These results indicate that CAMDI is localized at the proximal region of the leading process and required for dilation formation for radial migration during cortical development.

### CAMDI accumulates and oscillates at the dilation in cortical neurons during migration

The correlation between CAMDI localization and dilation formation suggests that neuronal migration is controlled by the change of the intracellular CAMDI expression and stabilization. Therefore, intensity of the EGFP-CAMDI expression in neuronal cells should change sequentially during migration. To examine the CAMDI localization linked to neuronal migration, the fluorescence intensity of EGFP-CAMDI was measured by time-lapse imaging. EGFP-CAMDI concentration is normalized to cytoplasmic volume (DsRed) along the entire length of migrating neurons. The EGFP-CAMDI enriched in the proximal region of the leading process (Fig. S2*A*). On the other hand, EGFP-CAMDI in the rear of the soma was not accumulated. We quantitated EGFP-CAMDI concentration that normalized to cytoplasmic volume (DsRed) in the different zones described above. EGFP-CAMDI accumulates in the dilation with a concurrent decrease in soma EGFP-CAMDI (Fig. S2, *B* and *C*). These findings indicated that changes in CAMDI concentration in the dilation occur before soma translocation during cortical migration.

**Figure 2:**
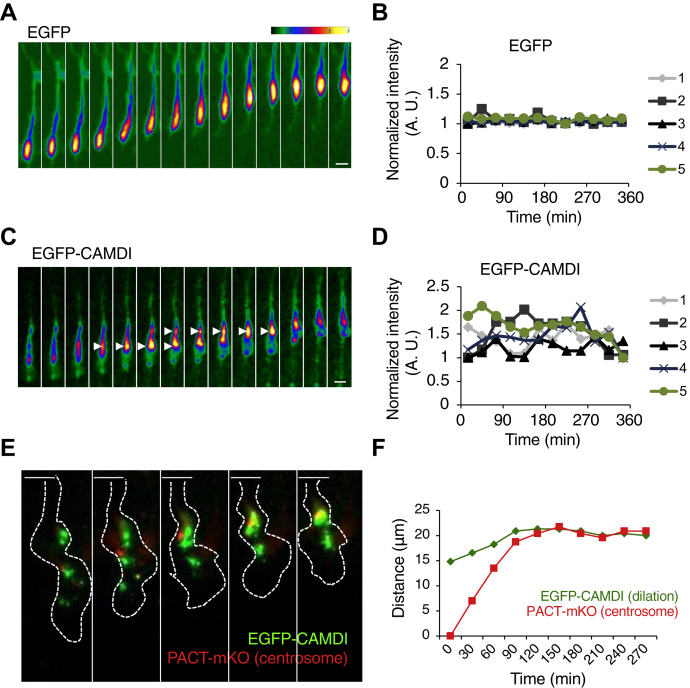
**CAMDI accumulates and oscillates at the dilation in cortical neurons during migration.***A*, the heat map represents the EGFP fluorescence intensity corresponding to time-lapse imaging of migrating neurons in cultured cortical slices. Coronal sections through the somatosensory cortex of E17.5 were analyzed after *in utero* electroporation at E14.5. EGFP-CAMDI localization is indicated. The image was taken every 15 min. The scale bar represents 10 μm. *B*, normalized intensity of EGFP electroporated migrating neuron. In addition to the cell in panel *A*, normalized intensities of five typical cells are drawn. *C*, the heat map represents the EGFP-CAMDI fluorescence intensity corresponding to time-lapse imaging of a migrating neuron. *Arrows* indicate the region of EGFP-CAMDI accumulation. The image was taken every 10 min. The scale bar represents 10 μm. *D*, normalized intensity of EGFP-CAMDI electroporated migrating neurons. In addition to the cell in panel *C*, normalized intensities of five typical cells are drawn. *E*, time-lapse imaging of EGFP-CAMDI (*green*) and PACT-mKO (centrosome, *red*) fluorescence during cortical migration. The image was taken every 30 min. The scale bar represents 10 μm. *F*, distance traveled of centrosome (*red*) to the dilation corresponding to EGFP-CAMDI (*green*) localization shown in panel *E*. CAMDI, coiled-coil protein associated with MRLC IIa and DISC1; E14.5, embryonic day 14.5; E17.5, embryonic day 17.5.

Although no change in fluorescence intensity was observed in the EGFP-expressing control neurons (Fig. 2*A*), it was revealed that the fluorescence intensity of EGFP-CAMDI–expressing cells repeatedly increased and decreased in an oscillating fashion (Fig. 2*C*). In addition, we noticed that, before neuronal migration, CAMDI accumulated in the cell soma and was enriched in the two proximal regions of the leading process. The two regions with the strongest fluorescence intensity coalesced together to form a single region with particularly strong EGFP-CAMDI intensity. Immediately after soma translocation, the intensity of EGFP-CAMDI returned to the same level as before migration (Fig. 2, *A* and *C* and Fig. S3*A*). The speed of neuronal migration is not affected by CAMDI overexpression (Fig. S3*B*). Quantitative analysis revealed that, while control EGFP-expressing neurons did not exhibit intensity changes, the fluorescence intensity of the EGFP-CAMDI–expressing neurons repeated the pattern of increase/decrease during the migration of neurons (Fig. 2, *B* and *D* and Fig. S4, *A*–*C*). It is well known that neuronal migration is achieved by sequential centrosome separation from the nucleus and nuclear movement. In our previous study, CAMDI was shown to regulate neuronal migration through regulating centrosome orientation, suggesting that CAMDI localized and regulated its stability at both the dilation and the centrosome during cortical migration. To investigate this, PACT-mKO, which visualized the centrosome, was expressed simultaneously together with EGFP-CAMDI, and their intensity was observed. EGFP-CAMDI localized around the centrosome, followed by accumulation at the dilation in the proximal region in the leading process (Fig. 2*E*). The centrosome and dilation signals were merged *via* centrosome movement to the dilation before soma translocation (Fig. 2*F* and Fig. S3*C*). These results indicated that the repetitive CAMDI oscillation at the dilation before soma translocation was required for cortical neuronal migration.

### Cdc20–APC/C–dependent ubiquitination and degradation of CAMDI

**Figure 3:**
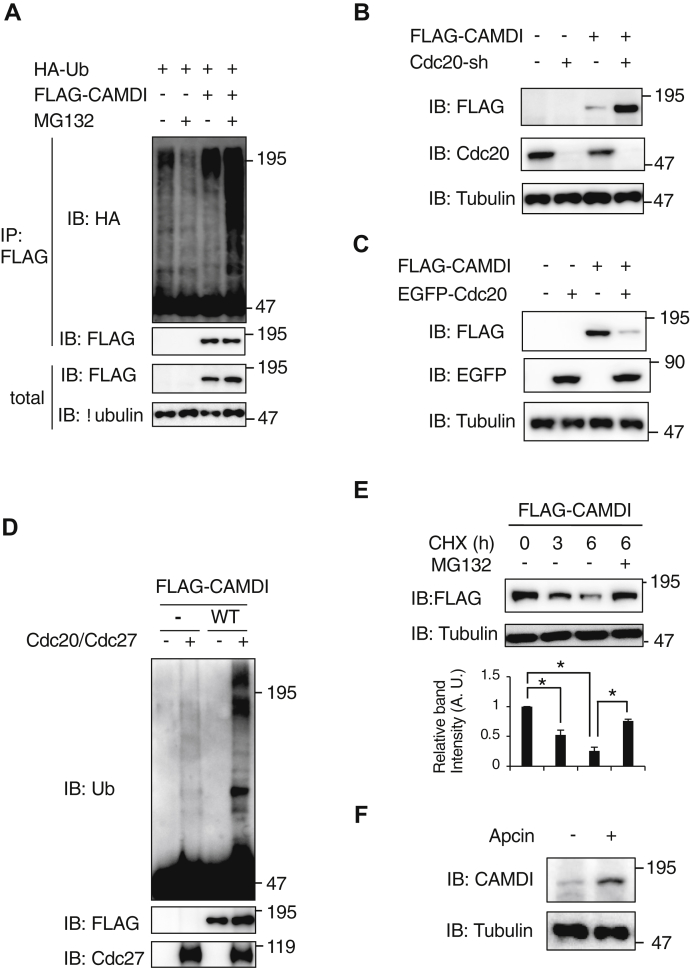
**CAMDI ubiquitination and degradation by the Cdc20–APC/C pathway.***A*, ubiquitination of CAMDI. HeLa cells were transfected with the indicated plasmids. Cells were collected after treatment with or without 50 μM MG132 for 3 h. The cell lysates were immunoprecipitated with the anti-FLAG antibody, followed by immunoblotting with the indicated antibodies. n = 3 independent experiments. *B*, Cdc20 knockdown induced CAMDI stabilization. HeLa cells were transfected with FLAG-CAMDI and Cdc20-sh plasmids. The cell lysate was subjected to Western blot analysis. n = 3 independent experiments. *C*, CAMDI was degraded by Cdc20 coexpression. HeLa cells were transfected with FLAG-CAMDI and EGFP-Cdc20 plasmids. The cell lysate was subjected to Western blot analysis. n = 3 independent experiments. *D*, *in vitro* ubiquitination assay. Immunoprecipitated FLAG-CAMDI was subjected to an *in vitro* ubiquitination assay. n = 3 independent experiments. *E*, effect of protein synthesis inhibitor CHX on CAMDI protein expression. HeLa cells expressing FLAG-CAMDI were treated with or without CHX (10 μg/ml) for the indicated times in the presence or absence of MG132 (50 μM), and cell lysates were immunoblotted with the anti-FLAG antibody. n = 3 independent experiments. ∗*p* < 0.05, one-way ANOVA with Bonferroni’s *post hoc* test. Data are presented as the mean ± SD. *F*, E14.5 brain slices were treated with or without Apcin (100 μM) for 6 h, and each lysate was subjected to immunoblot assay with the indicated antibody. n = 3 independent experiments. APC/C, anaphase-promoting complex/cyclosome; CAMDI, coiled-coil protein associated with MRLC IIa and DISC1; CHX, cycloheximide; E14.5, embryonic day 14.5; E17.5, embryonic day 17.5.

We examined what kind of molecular mechanism controls CAMDI stabilization. First, we found that CAMDI stability was regulated by the cell cycle machinery. The cell-cycle stage of FLAG-CAMDI–transfected HeLa cells were synchronized by double-thymidine block and released by thymidine washout. When cells entered the late S and G_2_/M phases ∼9 h after release, CAMDI expression was decreased (Fig. S5*A*). Transfected cells entered the M phase after 10 h, as determined by mitotic phosphorylation of histone H3 at Ser10. In addition, treatment with nocodazole, a drug known to destabilize microtubules, induced G_2_/M arrest and CAMDI degradation (Fig. S5*B*). We examined whether the CAMDI degradation occurred in a ubiquitin-dependent manner. FLAG-CAMDI and HA-Ub were cotransfected into HeLa cells treated with or without the proteasome inhibitor MG132. Cells were lysed and immunoprecipitated after immunoblot assay. We observed CAMDI polyubiquitination in the cultured cells (Fig. 3*A*). In addition, the level of CAMDI ubiquitination increased in the presence of proteasome inhibitor, MG132, indicating that CAMDI was degraded in a proteasome-dependent manner. This suggests that CAMDI is degraded through the G_2_/M ubiquitination system. It is well known that Cdc20–APC/C constitutes the Ub system of the G_2_/M phase. To investigate whether CAMDI stabilization was regulated through the Cdc20–APC/C system, we carried out a knockdown assay. HeLa cells were transfected with FLAG-CAMDI with or without shRNA against *Cdc20* gene plasmid and subjected to a Western blot assay. We observed that stabilization of FLAG-CAMDI occurred when cells were transfected with the Cdc20-sh plasmid, indicating that FLAG-CAMDI was degraded *via* Cdc20 (Fig. 3*B*). In contrast, FLAG-CAMDI was expressed in cultured cells with EGFP-tagged Cdc20 and analyzed by immuno precipitation-immuno blotting assay. CAMDI was degraded effectively by Cdc20 coexpression (Fig. 3*C*). *In vitro* ubiquitination assay revealed that immunoprecipitated FLAG-CAMDI was ubiquitinated in the absence of Cdc20 and immunoprecipitated Cdc27 complex (APC/C) (Fig. 3*D*). In addition, FLAG-CAMDI degradation was inhibited by MG132 treatment (Fig. 3*E*). The degradation of CAMDI was inhibited by treatment with an APC/C inhibitor, Apcin (Fig. 3*F*). These findings suggested that CAMDI was degraded by the Cdc20–APC/C pathway.

### CAMDI interacts with Cdc20–APC/C and colocalizes at the centrosome

**Figure 4:**
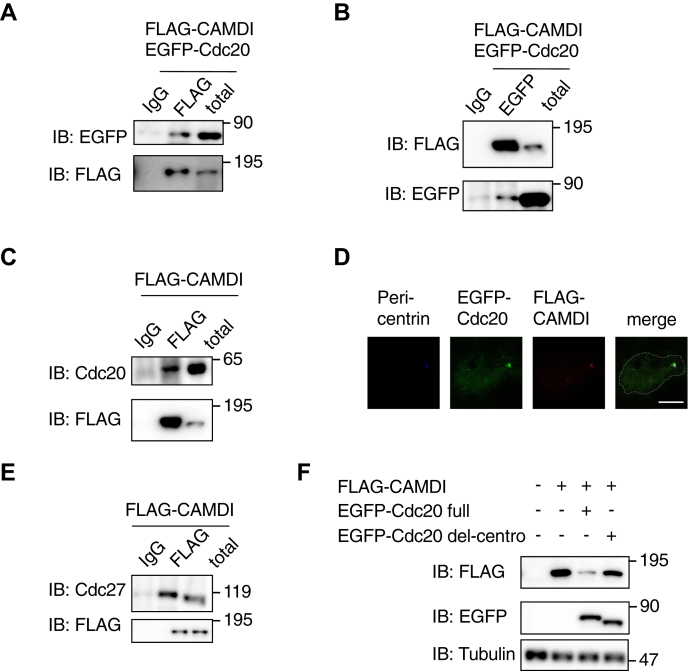
**CADMI interacts with Cdc20–APC/C at the centrosome.***A* and *B*, CAMDI interacted with Cdc20. HeLa cells were transfected with FLAG-CAMDI and EGFP-Cdc20 plasmids. Cell lysates were subjected to an immunoprecipitation assay with the anti-FLAG antibody (*A*) or anti-EGFP antibody (*B*) and immunoblot assay. n = 3 independent experiments. *C*, CAMDI interacted with endogenous Cdc20 in HeLa cells. Cells were transfected with the FLAG-CAMDI plasmid, followed by being subjected to immunoprecipitation and immunoblot assays. n = 3 independent experiments. *D*, CAMDI colocalized with Cdc20 at the centrosome. HeLa cells were transfected with FLAG-CAMDI and EGFP-Cdc20 plasmids. Cells were subjected to immunocytochemical analysis using anti-FLAG and anti-EGFP antibodies. Counterstaining with Hoechst was performed to visualize the nucleus. The scale bar represents 10 μm. *E*, CAMDI interacted with endogenous APC/C in HeLa cells. HeLa cells were transfected with the FLAG-CAMDI plasmid, followed by being subjected to immunoprecipitation and immunoblot assays. n = 3 independent experiments. *F*, CAMDI was degraded by Cdc20 but not by Cdc20 with deletion of the centrosome localization signal. HeLa cells were transfected with FLAG-CAMDI and EGFP-Cdc20 or EGFP-Cdc20 mutant with deletion of the centrosome localization signal. Cell lysates were subjected to immunoblot analysis. n = 3 independent experiments. APC/C, anaphase-promoting complex/cyclosome; CAMDI, coiled-coil protein associated with MRLC IIa and DISC1.

Because Cdc20–APC/C functions primarily by targeting key cell-cycle proteins for proteasomal degradation, we hypothesized that the interaction between CAMDI and APC/C may be important for the regulation of CAMDI’s protein level. We investigated whether CAMDI interacted with Cdc20–APC/C. When FLAG-CAMDI and EGFP-Cdc20 were cotransfected into HeLa cells, FLAG-CAMDI interacted with EGFP-Cdc20 (Fig. 4, *A* and *B*). FLAG-CAMDI also interacted with endogenous Cdc20 (Fig. 4*C*), whereas we could not detect an interaction and degradation of CAMDI *via* Cdh1, which also interacts with the APC/C ubiquitination system like Cdc20 (Fig. S6, *A*–*D*). In a previous report, Cdc20-APC/C was described as localizing at the centrosome and regulating its substrate degradation (22). In this study, CAMDI colocalized with Cdc20 at the centrosome (45.6 ± 9.1%; Fig. 4*D*). In cortical neurons, we confirmed that CAMDI interacted and colocalized with Cdc20 endogenously (8.6 ± 2.3%; Fig. S7, *A* and *B*). In addition, endogenous Cdc27, which is one of the subunits of APC/C, also coimmunoprecipitated with FLAG-CAMDI (Fig. 4*E*). Although Cdc20 has a centrosome-targeting sequence in its middle (22), EGFP-Cdc20 mutant, with deletion of this targeting sequence, could not degrade FLAG-CAMDI in cultured cells (Fig. 4*F*). These findings indicate that CAMDI interacted with Cdc20–APC/C at the centrosome and was degraded in a proteasome-dependent manner.

### Cdc20–APC/C–dependent degradation of CAMDI in a D-box–dependent manner

**Figure 5:**
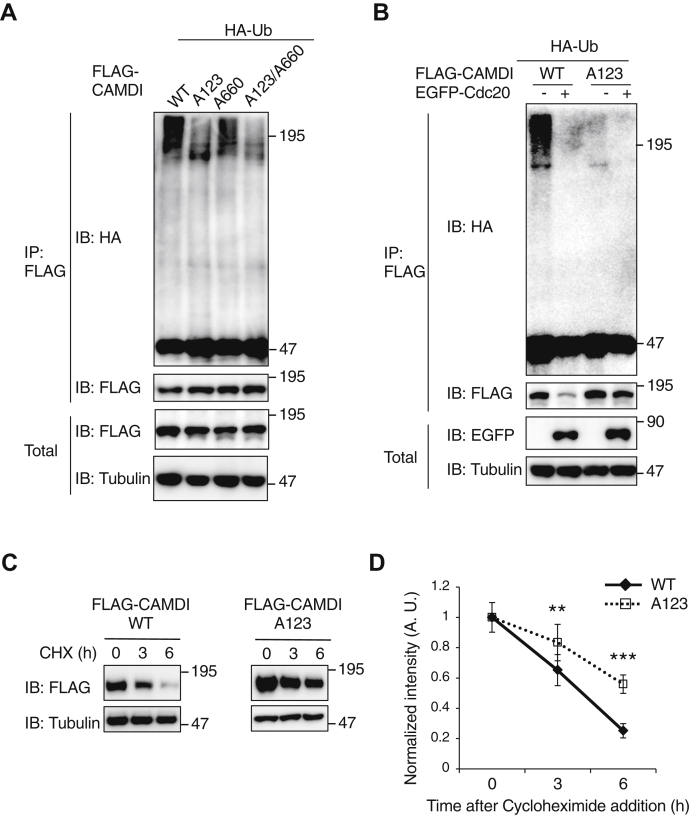
**Cdc20–APC/C–dependent degradation of CAMDI in a D-box–dependent manner.***A*, CAMDI was ubiquitinated in a D-box–dependent manner. HeLa cells were transfected with FLAG-CAMDI WT (full length), A123 (D-box at 123 aa), A660 (D-box at 660 aa), or A123/A660 double-mutant and HA-Ub plasmids. Cell lysates were immunoprecipitated with the anti-FLAG antibody and subjected to immunoblot assay. *B*, CAMDI D-box mutant was resistant to Cdc20-dependent ubiquitination. HeLa cells were transfected with FLAG-CAMDI WT or A123 with or without EGFP-Cdc20 plasmids and subjected to immunoblot assay. *C*, cycloheximide (CHX)-chase assay indicated the rapid degradation of CAMDI and resistance to degradation upon D-box mutation. HeLa cells transfected with the indicated plasmids were treated with CHX (10 μg/ml) for the indicated times, and each lysate was subjected to the immunoblot assay. *D*, the relative protein levels of FLAG-CAMDI in panel *C* were quantified by densitometry. n = 3 independent experiments. ∗∗*p* < 0.01; ∗∗∗*p* < 0.001, two-way ANOVA followed by Scheffe’s *post hoc* test. Data are presented as the mean ± SD. APC/C, anaphase-promoting complex/cyclosome; CAMDI, coiled-coil protein associated with MRLC IIa and DISC1; D-box, destruction box.

Cdc20 recognizes a specific sequence, called the D-box, so we analyzed the primary sequence of CAMDI for a potential D-box sequence and found that it has a D-box consensus sequence at 123 aa and 660 aa (RxxLxxL) (Fig. S8*A*). In this motif, two residues, an arginine and a leucine, are conserved in D-boxes in other Cdc20-binding proteins (28). We generated CAMDI mutants with replacement of arginine and leucine by alanine in the consensus sequence (AxxAxxL, designated A123, A660, and double-mutant A123/A660, Fig. S8*A*). CAMDI A123 mutant was found to be more resistant to degradation during the G_2_/M phase (Fig. S8*B*). WT CAMDI and A660 mutant were ubiquitinated, but CAMDI A123 and A123/A660 mutants were not (Fig. 5*A* and Fig. S9, *A* and *B*). We observed that ubiquitination and degradation of the CAMDI A123 and A123/A660 mutants were inhibited by Cdc20 coexpression (Fig. 5*B* and Fig. S9, *C* and *D*). Although the degradation of WT CAMDI and A660 was induced, CAMDI A123 and A123/A660 mutants were more resistant to degradation in cycloheximide-treated cells (Fig. 5, *C* and *D* and Fig. S9, *E* and *F*). These results suggested that CAMDI stabilization and degradation *via* Cdc20-APC/C were controlled in a D-box–dependent manner.

### Plk1-dependent phosphorylation and degradation of CAMDI

CAMDI degradation occurred during the G_2_/M phase, as determined by thymidine release assay (Fig. S8*B*). Mobility shift of CAMDI fragment 1 (1–250 aa) increased during nocodazole treatment, but not during thymidine treatment (Fig. S10*A*), suggesting that a post-translational modification, probably phosphorylation, regulated CAMDI stabilization. We hypothesized that the degradation of CAMDI was regulated by phosphorylation *via* G_2_/M kinase. It is well known that Plk1 is a critical molecule for cell-cycle regulation in the G_2_/M phase and is localized at the centrosome during this phase (29). In addition, we focused on Plk1 kinase because CAMDI has a consensus phosphorylation motif [D/E-*X*-S/T-x-*X*-D/E (*X*, any amino acid; x, a hydrophobic amino acid)] (30) for Plk1 in its N′ region, threonine 91. To investigate whether threonine 91 represents a phosphorylation site for Plk1, we carried out a band-shift assay. When threonine 91 was replaced with alanine of fragment 1 (1–250 aa T91A), no band shift was observed (Fig. S10*A*), suggesting that T91 represents a putative phosphorylation site by Plk1. Overexpression of HA-Plk1 reduced CAMDI expression (Fig. S10*B*). In addition, knockdown of Plk1 by shRNA against *Plk1*gene, in which the specificity was validated in a previous study (31), increased WT CAMDI expression, but not that of T91A mutant, indicating that Plk1 regulated CAMDI stabilization (Fig. S10*C*). Next, we investigated whether T91 phosphorylation influenced CAMDI degradation by Cdc20–APC/C. We constructed two full-length CAMDI mutants. One is a nonphosphorylated mutant, with mutation of threonine 91 to alanine (T91A). The T91A mutant shows resistance to Cdc20-dependent degradation (Fig. S10*D*). The other phosphor-mimetic mutant, with mutation of threonine 91 to glutamic acid (T91E), was not resistant to Cdc20-dependent degradation, like WT CAMDI (Fig. S10*D*). Although T91E CAMDI was also degraded as well as the WT CAMDI in cycloheximide-treated HeLa cells, the T91A mutation gave stability against Cdc20-dependent degradation (Fig. S10, *E* and *F*). These findings suggested that phosphorylation of CAMDI at T91 by Plk1 affected CAMDI degradation.

### Dilation formation and radial migration through oscillation of CAMDI stabilization in a Cdc20–APC/C–dependent manner

**Figure 6:**
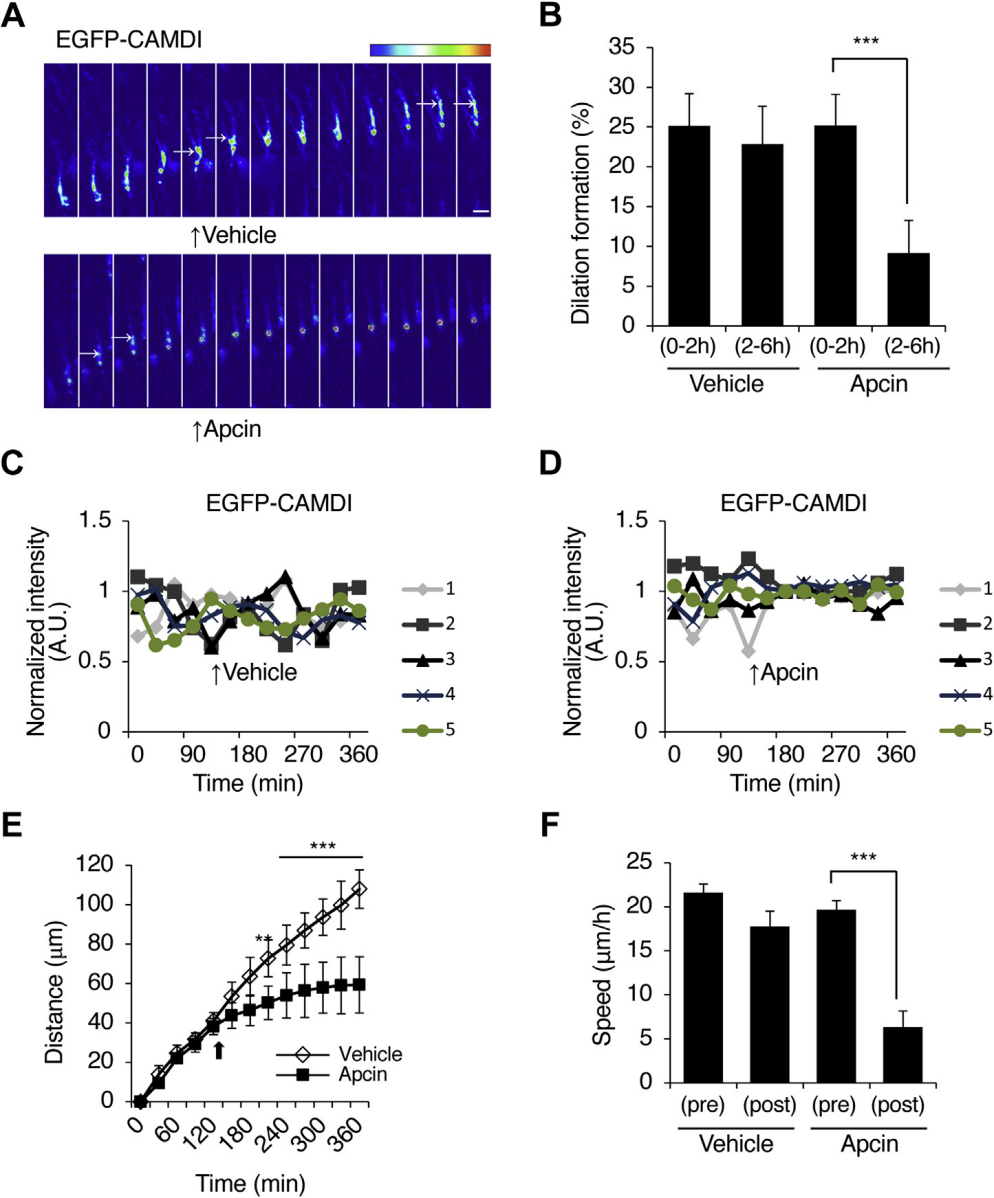
**Dilation formation and radial migration through oscillation of CAMDI in a Cdc20-APC/C–dependent manner.***A*, representative heat map images of EGFP-CAMDI electroporated neuron treated with or without APC/C inhibitor, Apcin (100 μM). Coronal sections through the somatosensory cortex of E17.5 were analyzed after *in utero* electroporation of EGFP plasmid at E14.5 using time-lapse imaging analysis. The image was taken every 30 min. The scale bar represents 10 μm. *B*, percentage of dilation formation shown in panel *A*. Apcin treatment causes inhibition of dilation formation. n = 3 mice/group (vehicle = 135 cells, Apcin = 157 cells). ∗∗∗*p* < 0.001, two-way ANOVA followed by Scheffe’s *post hoc* test. Data are presented as the mean ± SD. *C* and *D*, EGFP-CAMDI intensity traces of five typical migrating neurons with vehicle (*C*) and Apcin (*D*) treatment. Oscillation of EGFP-CAMDI fluorescence intensity during neuronal migration disappeared upon the addition of Apcin. *E*, migrating distance of vehicle- or Apcin-treated neurons. n = 3 mice/group (vehicle = 10 cells, Apcin = 10 cells). ∗∗*p* < 0.01; ∗∗∗*p* < 0.001, two-way ANOVA followed by Scheffe’s *post hoc* test. Data are presented as the mean ± SD. *F*, migration speed of vehicle- or Apcin-treated neurons. n = 3 mice/group (vehicle = 10 cells, Apcin = 10 cells). ∗∗∗*p* < 0.001, two-way ANOVA followed by Scheffe’s *post hoc* test. Data are presented as the mean ± SD. APC/C, anaphase-promoting complex/cyclosome; CAMDI, coiled-coil protein associated with MRLC IIa and DISC1; E14.5, embryonic day 14.5; E17.5, embryonic day 17.5.

The results showing that the stability of CAMDI was regulated by the Cdc20–APC/C cell cycle machinery suggested the possibility that the mode of locomotion for neuronal migration during development is regulated by stable/unstable oscillation of CAMDI. To investigate whether Cdc20–APC/C functions in dilation formation and radial neuronal migration *in vivo*, we analyzed EGFP-CAMDI electroporated brain slices using a live-imaging assay with or without treatment with an APC/C inhibitor, Apcin. In a control experiment, EGFP-CAMDI–expressing cells had dilation at a rate of about ∼25% both before (0–2 h) and after (2–6 h) DMSO treatment (Fig. 6, *A* and *B*). On the other hand, the proportion of migrating neurons with dilation formation was decreased (∼5%) after Apcin treatment (2–6 h) (Fig. 6, *A* and *B*). In addition, Apcin treatment impaired the stable/unstable oscillation of EGFP-CAMDI (Fig. 6, *C* and *D* and Fig. S11, *A* and *B*) and inhibited radial migration (Fig. 6, *E* and *F*). These findings indicated that APC/C regulates cortical neuronal migration through dilation formation by the oscillation of CAMDI stabilization *via* the Cdc20–APC/C system.

### Oscillation of CAMDI stable/unstable states is required for proper dilation formation and radial migration

**Figure 7:**
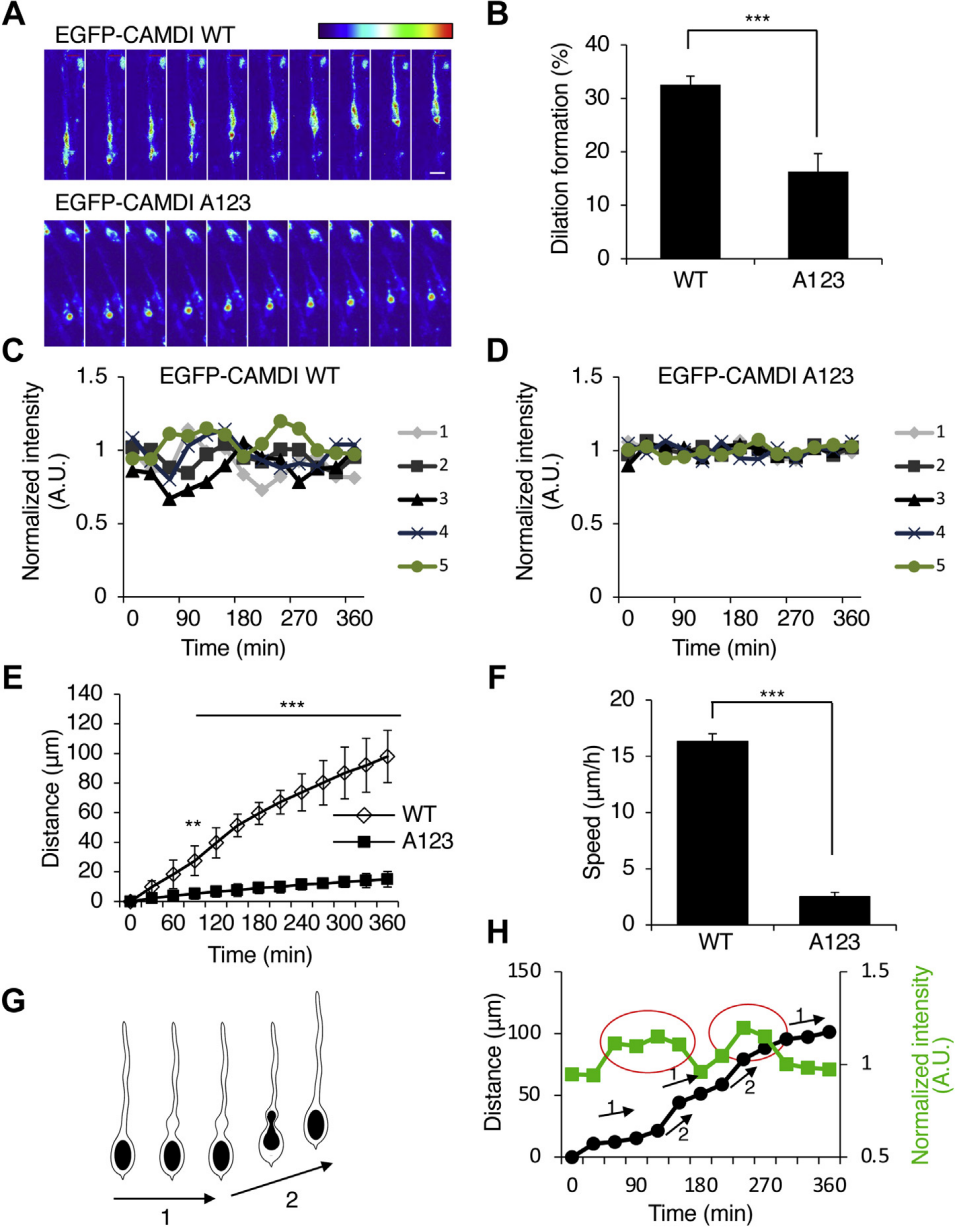
**Oscillation of CAMDI stable/unstable states is required for proper dilation formation and radial migration.***A*, representative heat map images of single EGFP-CAMDI WT or EGFP-CAMDI A123 plasmid electroporated neurons. Coronal sections through the somatosensory cortex of E17.5 were analyzed after *in utero* electroporation of EGFP plasmid at E14.5 using time-lapse imaging analysis. The image was taken every 30 min. The scale bar represents 10 μm. *B*, percentage of dilation formation shown in panel *A*. EGFP-CAMDI A123 expression causes inhibition of dilation formation. n = 3 mice/group (WT = 104 cells, A123 = 90 cells). ∗∗∗*p* < 0.001, one-way ANOVA with Bonferroni’s *post hoc* test. Data are presented as the mean ± SD. *C* and *D*, EGFP-CAMDI intensity traces of five typical migrating neurons electroporated with WT (*C*) and A123 (*D*) plasmid. Oscillation of EGFP-CAMDI fluorescence intensity during neuronal migration disappeared upon EGFP-CAMDI A123 expression. *E*, migration distance of EGFP-CAMDI WT or A123 electroporated neurons. n = 3 mice/group (WT = 10 cells, A123 = 10 cells). ∗∗*p* < 0.01; ∗∗∗*p* < 0.001, two-way ANOVA followed by Scheffe’s *post hoc* test. Data are presented as the mean ± SD. *F*, migration speed of EGFP-CAMDI WT or A123 electroporated neurons. n = 3 mice/group (WT = 10 cells, A123 = 10 cells). ∗∗*p* < 0.01; ∗∗∗*p* < 0.001, one-way ANOVA with Bonferroni’s *post hoc* test. Data are presented as the mean ± SD. *G*, schematic diagram of a migrating neuron. Dilation formation states (1) and cell soma translocation states (2). *H*, graphical representation of the change in intensity of EGFP-CAMDI and cell soma translocation. EGFP-CAMDI stabilization (*red circle*) was linked to cell soma translocation states [(1, 2) in panel *G*]. CAMDI, coiled-coil protein associated with MRLC IIa and DISC1; E14.5, embryonic day 14.5; E17.5, embryonic day 17.5.

**Figure 8:**
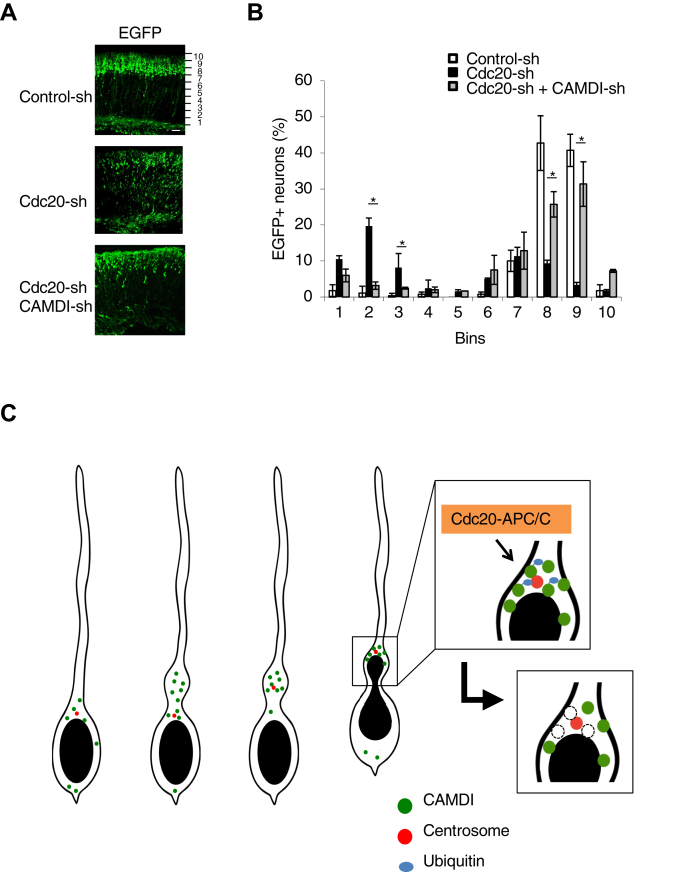
**Functional interaction between CAMDI and Cdc20.***A*, sagittal sections of mouse brains electroporated at E14.5 *in utero* with indicated plasmids plus EGFP were analyzed at P2. The scale bar represents 100 μm. *B*, quantification of the number of EGFP-positive neurons. n = 3 mice/genotype (control-sh = 608 cells, Cdc20-sh = 895 cells, Cdc20-sh + CAMDI-sh = 684 cells). *Asterisks* indicate significant differences between Cdc20-sh and Cdc20-sh + CAMDI-sh. ∗*p* < 0.05; two-way ANOVA followed by Scheffe's *post hoc* test. Data are presented as the mean ± SD. *C*, schema of dilation formation by CAMDI. CAMDI, coiled-coil protein associated with MRLC IIa and DISC1; CAMDI-sh, shRNA against mouse *Ccdc141* gene that encodes CAMDI; E14.5, embryonic day 14.5.

To demonstrate that the degradation of CAMDI by APC/C is important for neuronal migration, we investigated whether the expression of A123 CAMDI induced abnormal dilation formation and neuronal migration *in vivo*. The results revealed that neurons expressing A123 CAMDI exhibited reduced dilation formation (Fig. 7, *A* and *B*). Although EGFP-CAMDI repeated the stable/unstable states in cortical migration (Fig. 7*C* and Fig. S11*C*), the oscillation disappeared in EGFP-CAMDI A123 electroporated neurons (Fig. 7*D* and Fig. S11, *D* and *E*). These neurons showed delayed radial migration with decreases of migration distance and speed (Fig. 7, *E* and *F*). The distance traveled by cell soma was correlated with CAMDI stabilization, suggesting that the stabilization of CAMDI accompanied and promoted cell soma translocation (Fig. 7, *G* and *H*). To investigate a functional interaction between CAMDI and Cdc20, histological analyses of radial migration were performed by *in utero* electroporation assay. In control-sh electroporated mice, almost all EGFP^+^ cells electroporated at E14.5 migrated to layers II/III of the cerebral cortex at P2. In contrast, several EGFP^+^ cells in Cdc20-sh electroporated mice remained in the lower cortical layers (Fig. 8*A*). This effect of Cdc20-sh was restored by a cotransfection of the CAMDI-sh (Fig. 8*B*). These results indicate an epistasis interaction between CAMDI and Cdc20 in cortical neurons.

## Discussion

In this study, live imaging of EGFP-CAMDI and the centrosome (PACT-mKO) revealed that CAMDI is involved in dilation formation by localizing at the proximal region of the leading process ahead of the centrosome. CAMDI localizes at the centrosome in a Disrupted in Schizophrenia 1–dependent manner and functions for centrosome maturation *via* negative regulation of tubulin deacetylase HDAC6 (23, 25), but its function outside the centrosome was unknown. From the results of knockdown experiments of CAMDI, CAMDI is necessary for dilation formation. In addition, signals of both EGFP-CAMDI at the centrosome and dilation, to which the centrosome will translocate in the future, were merged *via* centrosome movement to the dilation. The dilation serves as a region for anchoring the centrosome and may function as a mechanical structure for pulling up the cell soma using microtubules. It is considered that CAMDI promotes microtubule acetylation, which in turn promotes stabilization of the dilation and maturation of the centrosome. Soma translocation occurred during the stable phase of EGFP-CAMDI. In the subsequent process, the movement of neuronal cell soma was completed and progressed again to the next unstable phase of CAMDI, which returned to the same level of intensity as before migration. These new findings provide the first evidence that CAMDI controls a novel mechanism of neuronal migration by controlling dilation formation and that CAMDI stable/unstable oscillation is necessary for shifting to the next cycle of neuronal migration. The contribution of CAMDI in zone I is a subject for future work. To make the conclusion more certain, it will be important to detect that endogenous CAMDI undergoes similar oscillation.

Surprisingly, the CAMDI degradation was carried out *via* a proteasome-dependent system defined by Cdc20–APC/C for mitotic function. It is reported that a centrosomal Cdc20–APC pathway controls dendrite morphogenesis in postmitotic neurons (22). In addition, it was shown that APC/C functions in neurogenesis, glial differentiation and migration, neuronal survival and metabolism, neuronal morphogenesis, synapse formation and plasticity, and learning and memory (32). Our results indicated that Cdc20 degraded CAMDI at both the centrosome and the dilation after centrosome translocation to the dilation. Through this degradation, the next new dilation formation occurred to reset the oscillation of CAMDI stabilization, with subsequent cortical formation being accomplished by repeating these processes in migrating neurons. From the experiments using CAMDI A123, a mutant of the site recognized by Cdc20–APC/C, and the experiment using Apcin, which is an inhibitor of APC/C, the importance of regulation of the CAMDI stabilization cycle in these sequential processes was confirmed by degradation of CAMDI through Cdc20–APC/C for dilation formation during development. In this study, the EGFP-CAMDI plasmid was electroporated to neurons without knockdown or KO of endogenous CAMDI, which indicated that the increase of CAMDI protein level is manipulated by overexpression of EGFP-CAMDI. Despite the CAMDI protein level being manipulated to overexpression, the distance and speed of neuronal migration were unaffected. These data indicate that the activity of endogenous Cdc20–APC/C is sufficient even for degradation of overexpressed CAMDI protein, so that they are not affected by CAMDI overexpression. This is the first evidence that the neuronal migration was controlled by the Cdc20–APC/C degradation system, which regulated the oscillation of CAMDI stabilization for dilation formation and centrosome translocation (Fig. 8*C*).

In our previous study, HDAC6 hyperactivation by CAMDI deletion was shown to cause psychiatric behaviors through delayed radial migration because of impaired centrosomes (25). The Cdc20–APC/C system regulates the cell cycle of neural progenitor cells and the dendrite morphogenesis in postmitotic neurons, suggesting that the system controls the neuronal migration between premitotic and postmitotic neurons during cortical development. In addition, the centrosome-associated HDAC6 promotes the polyubiquitination of Cdc20, stimulating the activity of centrosomal Cdc20–APC/C (22). These findings indicated that CAMDI inhibited Cdc20–APC/C activity *via* HDAC6 inhibition, suggesting that CAMDI stabilization was regulated positively by a feedback mechanism *via* Cdc20–APC/C inhibition at the centrosome. However, the mechanism controlling the Cdc20–APC/C activity to induce ubiquitination remained unclear. In this study, Plk1 phosphorylated CAMDI at threonine 91 and accelerated CAMDI degradation (Figure 3, Figure 4, Figure 5), suggesting that dilation formation and centrosome movement during cortical migration might be controlled by molecular components for G_2_/M phase transition.

The cell cytoskeleton is critical for neuronal migration. Abnormal cytoskeleton-related genes are known as the cause of the onset of psychiatric disorders (26, 33). It was reported that the actin cytoskeleton was localized in the vicinity of the centrosome of migrating neurons (11, 16, 34, 35, 36). It is suggested that not only the control of the microtubule structure but also that of the actin cytoskeleton may be involved in the dilation formation and centrosome movement into the dilation. It was shown that the actin cytoskeleton of the tailing process behind the migrating cell soma is a factor necessary for extrusion of the nucleus and cell soma (15, 37). CAMDI controls the stabilization of the centrosome through negative regulation of HDAC6 activity and binds to active MRLC at the centrosome (23, 25), suggesting that CAMDI coordinated the dilation formation and centrosome translocation into the dilation *via* controlling both the actin and the microtubule cytoskeletons. More analysis is required to show whether perturbed microtubule/actin organization or dynamics contribute to the loss of dilation formation apart from effects on the centrosome. Further studies are needed to clarify the detailed molecular mechanism underlying regulation of the actin cytoskeleton by CAMDI.

## Experimental procedures

### Plasmids

Mouse full-length FLAG-CAMDI was constructed as in a previous study (23). FLAG-CAMDI A123, A660, A123/A660, T91A, and T91E were generated by QuikChange mutagenesis. To construct a plasmid for EGFP-CAMDI, the PCR product of CAMDI from RT-PCR was inserted into the SalI/BamHI sites of pEGFP-C1. For *in vivo* live imaging, EGFP-CAMDI created by standard PCR subcloning into the XhoI/BglII sites of pCAGGS using EGFP-CAMDI as the template. To construct a plasmid for EGFP-Cdc20, the PCR product of Cdc20 from RT-PCR was inserted into the EcoRI/SalI sites of pEGFP-C1. EGFP-tagged Cdc20 deletion expression plasmid was created by standard PCR subcloning into pEGFP-C1 using Cdc20-WT as the template (22). Several shRNA-targeted sequences are shown below (with an order of sense, loop (underlined), and antisense): control-sh (5′-ACTACCGTTGTATAGGTGTTCAAGAGACACCTATAACAACGGTAGT-3′), CAMDI-sh (5′-GGGTAGCCTATAATGACAAGCTTCAAGAGAGCTTGTCATTATAGGCTACCC-3′) (23), Cdc20-sh (5′-AACACCATGTGGCCACA CTTTCAAGAGAAGTGTGGCCACATGGTGTT-3′) (22), and Plk1-sh (5′-CGGCAGCGTGCAGATCAACTTCAAGAGAGTTGATCTGCACGCTGCCG-3′) (38). To construct a plasmid for shRNA, two primers were annealed, and the product was inserted into BamHI/HindIII sites of pSilencer 3.1-H1.

### Antibodies

Anti-FLAG M2 monoclonal, anti-actin, and anti-α-tubulin antibodies were obtained from Sigma-Aldrich. The anti-HA high-affinity antibody was obtained from Roche Applied Science. Anti-GFP rabbit polyclonal antibody and secondary antibodies conjugated with Alexa Fluor 350, 488, and 594 were obtained from Invitrogen. Anti-GFP mouse monoclonal and polyclonal antibodies were purchased from Clontech. Anti-Cdc20, anti-Cdc27, and anti-Ub (P4D1) antibodies were obtained from Santa Cruz Biotechnology. Antiphosphohistone H3 antibody was obtained from Millipore.

### Cell culture, transfection, and synchronization

HeLa cell line was obtained and authenticated from the American Type Culture Collection. Their identity was assumed to be determined by the American Type Culture Collection. Cells were routinely tested for *Mycoplasma* every 6 months. The cells were maintained in Dulbecco's modified Eagle's medium supplemented with 10% fetal bovine serum at 37 °C, in 5% CO_2_, in a humidified chamber. Transfection was carried out using Lipofectamine 2000 (Invitrogen). HeLa cells were cultured and synchronized by double-thymidine block or nocodazole treatment.

### *In vitro* ubiquitination assay

Immunoprecipitated FLAG-CAMDI was prepared from the lysate of HeLa cells transfected with the FLAG-CAMDI plasmid. Immunoprecipitated endogenous Cdc20 was prepared from the lysate of HeLa cells synchronized at the G2/M phase by nocodazole. The APC/C was purified using anti-Cdc27 from synchronized HeLa cells at G2/M. Immunoprecipitates were incubated with the reaction buffer (50 mM Tris, pH 7.4, 2 mM MgCl_2_, 4 mM ATP,100 ng E1 (Biomol), 300 ng UbcH10 (Biomol), and 2 μg His-ubiquitin (Biomol)) for 2 h at 30 °C and then terminated with the sample buffer.

### Immunoprecipitation and Western blotting

Culture cells were lysed in Nonidet P-40 lysis buffer (20 mM Tris HCl, pH 7.2, 2 mM EDTA, 0.5% Nonidet P-40, 8% sucrose, 80 mM dithiothreitol). The lysate was clarified by centrifugation at 15,000*g* for 10 min and immunoprecipitated with the appropriate antibody. Immunoprecipitates were washed three times with the lysis buffer. After boiling for 3 min, equal protein amounts of the lysates were subjected to SDS-PAGE and transferred to polyvinylidene difluoride membranes (Immobilon P; Millipore). Membranes were blocked for 1 h at room temperature (RT) in 5% skim milk in PBST with gentle shaking and incubated with primary antibodies overnight at 4 °C. After washing the membranes three times with PBST, they were incubated with the secondary antibody conjugated to horseradish peroxidase for 1 h at RT. The blotted membranes were developed using the Immobilon Western chemiluminescent HRP substrate (Millipore) according to the manufacturer's instructions.

### Immunocytochemistry

Cells were fixed for 20 min in PBS containing 4% paraformaldehyde and permeabilized with 0.2% Triton X-100. After incubation in PBS containing 1% bovine serum albumin for 30 min, the cells were reacted with the first antibody for overnight at 4 °C, followed by incubation with the secondary antibody. The staining was analyzed by a confocal microscope (Olympus FV1000-D).

### Immunohistochemistry

For immunohistochemical analysis, brains were fixed by immersion in 4% paraformaldehyde in 0.1 M PBST (PBS containing 0.1% Tween-20), cryoprotected in 20% sucrose, frozen in OCT compound, and stored at −80 °C until sectioning. Coronal sections (20 μm) were cut with a cryostat and stored at −20 °C before use. Tissue sections were blocked for 1 h at RT in PBS containing 5% horse serum and then incubated overnight at 4 °C with the primary antibody. For analysis of neuronal morphology and dendrite spine number *in vivo*, three-dimensional reconstructions of each EGFP-positive neuron were produced by Z-series stacks of confocal images. The projection images were automatically traced with ImageJ software (National Institutes of Health).

### *In utero* electroporation

*In utero* electroporation was performed as described previously (23). Briefly, *in utero* electroporation to the dorsal neocortex was performed by injecting the DNA plasmid solution (5 mg/ml) plus 1% Fast Green using a glass capillary into the E14.5 ICR mice ventricle. DNA mixture was 2- to 3-fold higher than that of the EGFP plasmid, which was the electroporation marker. Electroporation was performed using a CUY-21 electroporator (NEPA GENE) and the following parameters: four 50-ms-long pulse separated by 950-ms-long intervals at 33 V.

### Live imaging

For live imaging, coronal slices (200-μm thick) were prepared 48 to 72 h after *in utero* electroporation and then cultured on a Millicell Cell Culture Insert (Millipore) submerged in the Neurobasal medium (Invitrogen) supplemented with 10% fetal bovine serum, 1 mM glutamine, and 2% B-27 (Invitrogen). Electroporated neurons were imaged on an inverted microscope FV1000-D (Olympus) and BZ-X800 (Keyence). Sections were obtained and stacked to acquire whole images and collected every 10 min for 3 to 6 h.

### Image analysis

The images were analyzed using FIJI software. For distribution of EGFP-CAMDI in neurons, a line (30 pixels), which encompassed the entire width of the single neuron, was drawn from the rear of the soma to the distal region of the leading process. The intensity along the length of the line was measured and calculated from the proportion of each zone to the total fluorescence intensity. The intensity of the fluorescence signal is shown as a heat map (pseudocolor, intensity increases from green to yellow according to the scale). Dilation was defined as a swelling in the leading process within 20 μm from the tip of the nucleus during time-lapse observation. For volumetric analysis, region of interest were set up within each zone and measured of fluorescence intensity and the volume was obtained from stacked image sequence using 3D Object Counter (FIJI plug-in).

CAMDI levels was normalized to each volume during phase 1 and phase 2. All the fluorescence images were background-subtracted before quantification.

### Ethics statement

All animals were maintained under the university guidelines for the care and use of animals. The experiments were performed after securing Tokyo University of Pharmacy and Life Sciences Animal Use Committee Protocol approval.

### Statistical analyses

All results are expressed as the mean ± SD. For cell quantifications and behavioral tests, either one-way ANOVA with Bonferroni's *post hoc* test or two-way ANOVA with repeated measures followed by Scheffe's *post hoc* test was used.

## Data availability

All data are contained within the article.

## Supporting information

This article contains supporting information.

## Conflict of interest

The authors declare that they have no conflicts of interest with the contents of this article.
