# Supplementary material for: Oscillation of Cdc20–APC/C–mediated CAMDI stability is critical for cortical neuron migration
Source: J Biol Chem. 2021 Jul 21;297(2):100986. doi: 10.1016/j.jbc.2021.100986 (PMC8353494; doi:10.1016/j.jbc.2021.100986)
Supplement: Supplemental Figures S1–S11 [file mmc1.pdf]

Supporting Information

Oscillation of Cdc20-APC/C-mediated CAMDI stability is critical for cortical neuron migration

Shohei Okuda[1,3], Mariko Sato[1,3], Saho Kato[1,3], Shun Nagashima[1], Ryoko Inatome[2], Shigeru Yanagi[2] and Toshifumi Fukuda[1,4]

Figure S1

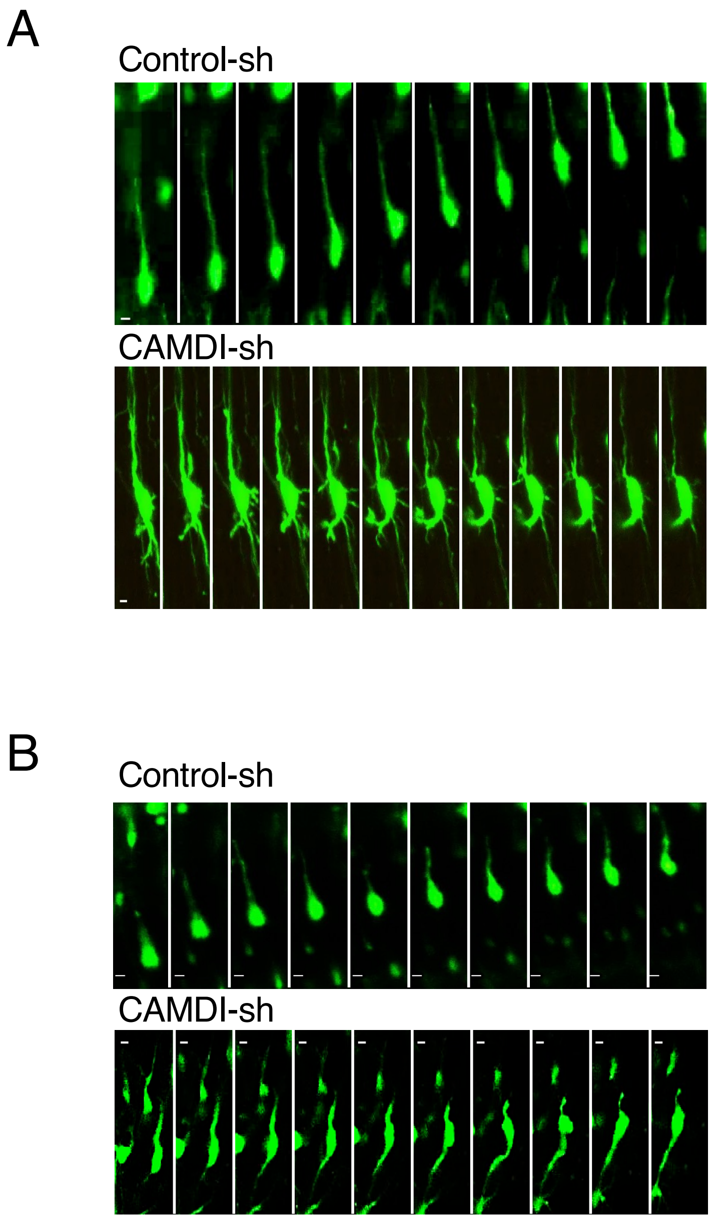

Figure S1. CAMDI knockdown inhibits dilation formation and radial migration (A, B) Another example of CAMDI-sh electroporated neurons. Image taken every 30 minutes. Scale bar, 5 μm.

Figure S2

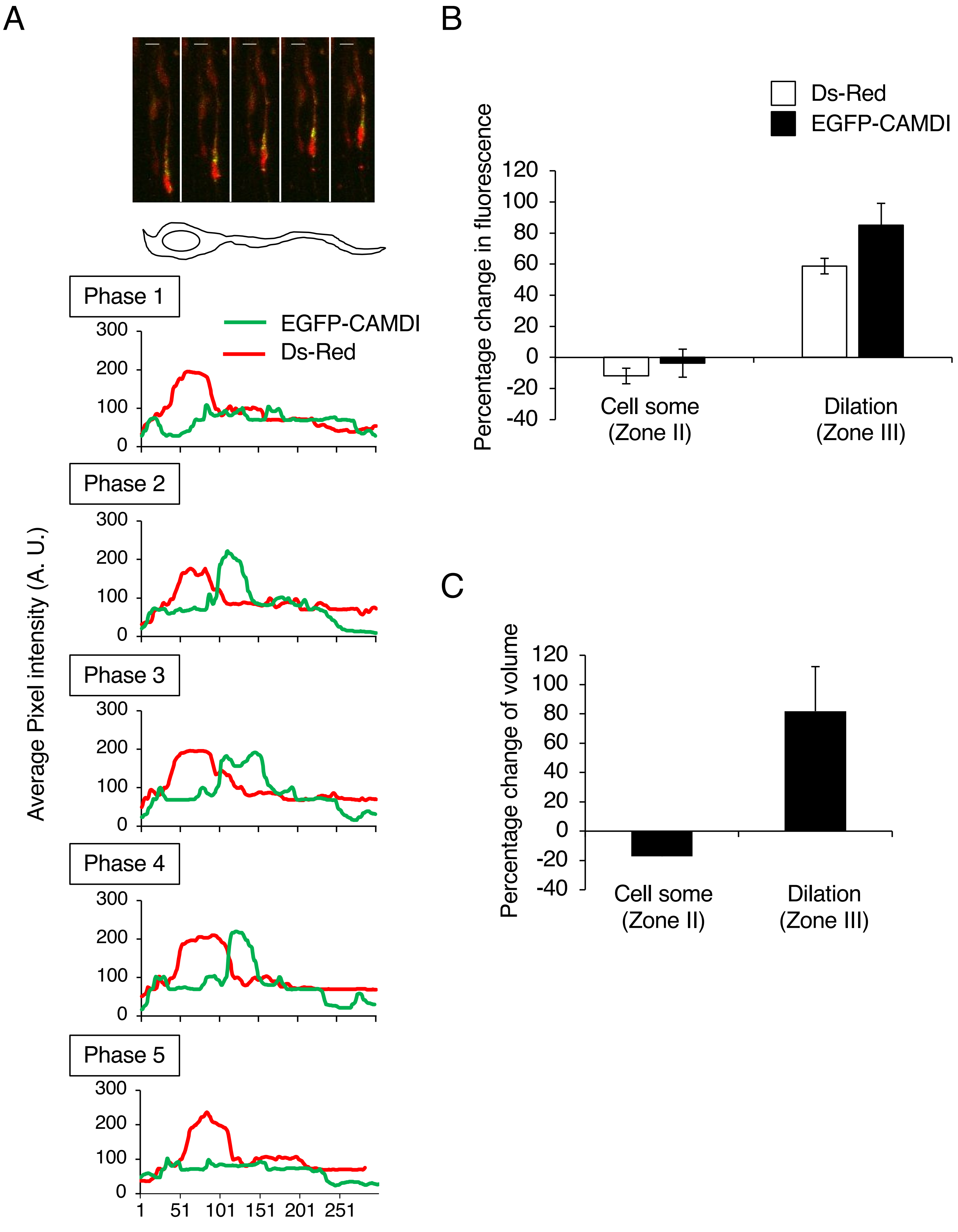

A
B
C
Phase 1
Phase 2
Phase 3
Phase 4
Phase 5
EGFP-CAMDI
Ds-Red
Average Pixel intensity (A. U.)
300
200
100
0
1
51
101
151
201
251
Percentage change in fluorescence
120
100
80
60
40
20
0
-20
-40
Cell some
(Zone II)
Dilation
(Zone III)
Percentage change of volume

Figure S2. Line scan and volumetric analysis of EGFP-CAMDI in migrating neurons

(A) Representative images and line-scan data of EGFP-CAMDI and Ds-Red plasmids electroporated neuron. Coronal sections through the somatosensory cortex of E17.5 were analyzed following in utero electroporation at E14.5. Line scan of the images shown in top panel to illustrate the distribution of EGFP-CAMDI and the cytoplasm (Ds-Red). Image taken every 30 minutes. Phase 1: pre-accumulation. Phase 2: EGFP-CAMDI accumulation at dilation. Phase 3: EGFP-CAMDI accumulation at dilation and centrosome. Phase 4: The two regions coalesced together to form a single region of EGFP-CAMDI. Phase 5: EGFP-CAMDI returned to the same level as phase 1.

(B, C) Summary of changes in protein/cytoplasmic concentration and cell volume during phase 1 and phase 2 (n = 5). There was a 3.7% (±9%) decrease in EGFP-CAMDI concentration, an 12% (±7%) decrease in cytoplasmic concentration (Ds-Red), and a 17% (±6%) decrease in overall volume of the cell soma during this period. In the proximal region of the leading process, the EGFP-CAMDI concentration increased by 85% (±14%), accompanying a 59% (±22%) increase in the cytoplasm concentration, and a 82% (±30%) increase in the overall volume of the proximal region.

Figure S3

A

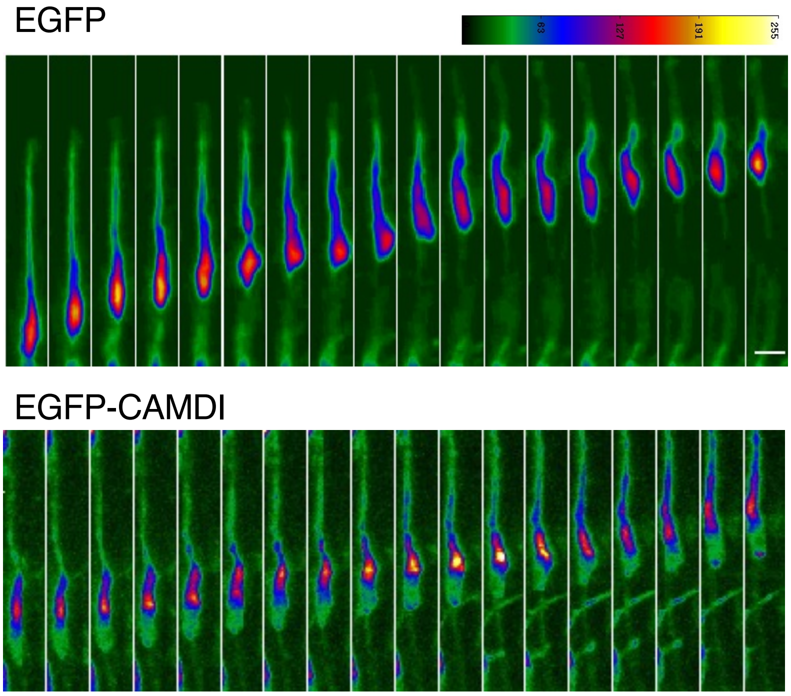

B

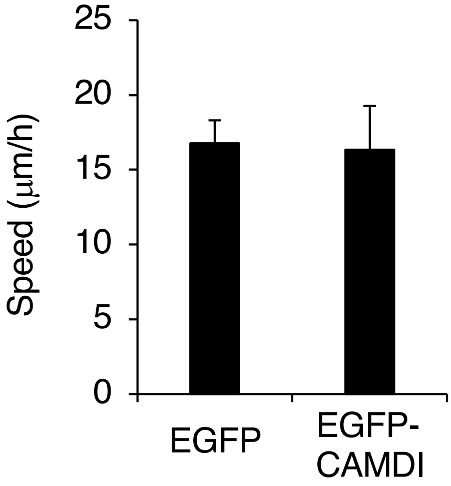

C

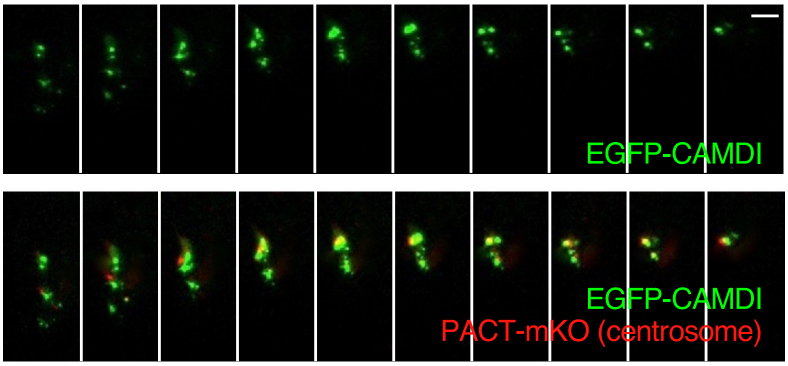

Figure S3. Oscillation of EGFP-CAMDI fluorescence intensity during cortical neuronal migration

(A) Heat map image of EGFP-CAMDI electroporated neurons. Image taken every 10 minutes. Scale bar, 10 μm.

(B) Migration speed of EGFP or EGFP-CAMDI electroporated neurons. n = 3 mice/ group (EGFP = 10 cells, EGFP-CAMDI = 10 cells). N. S., not significant. one-way ANOVA with Bonferroni's *post hoc* test. Data are presented as mean ± SD.

(C) Time-lapse imaging of EGFP-CAMDI (green) and PACT-mKO (centrosome, red) fluorescence during cortical migration. Weak magnification of Figure 2E. Image taken every 30 minutes. Scale bar 10 μm.

Figure S4

A

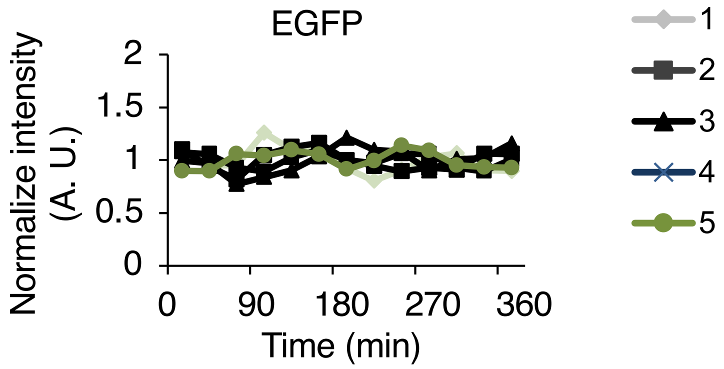

B

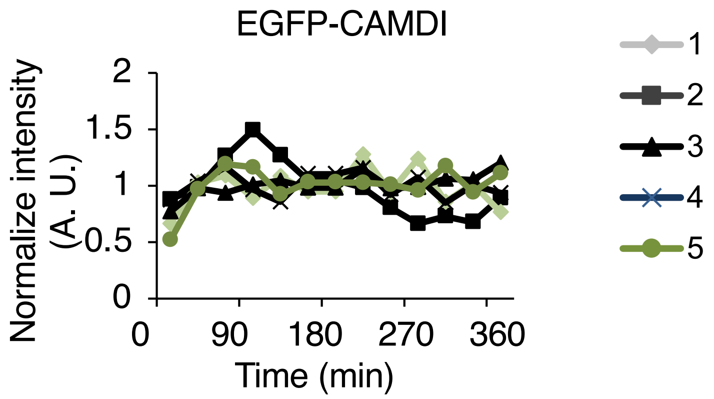

C

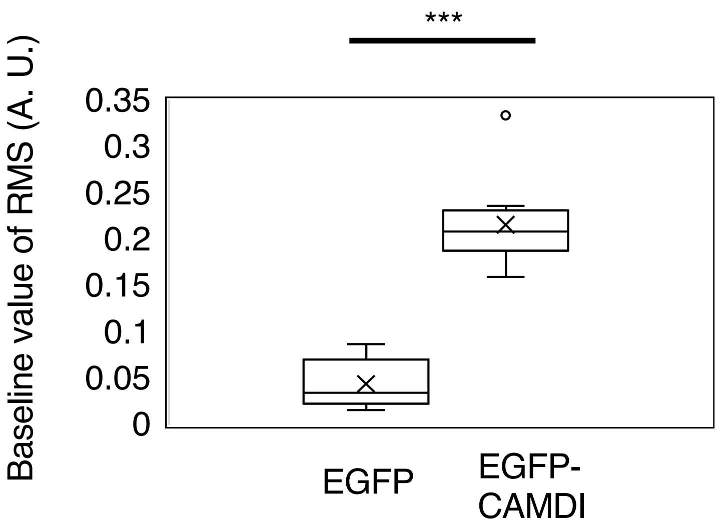

Figure S4. Normalized intensity and Box-and-whisker plot analysis of electroporated migrating neuron

(A, B) Normalized intensity of EGFP (A) and EGFP-CAMDI (B) related to Figure 2B and D.

(C) Box-and-whisker plot analysis of Baseline value of the RMS (the root mean square). N=10 cells. ***, $p<0.001$.

Figure S5

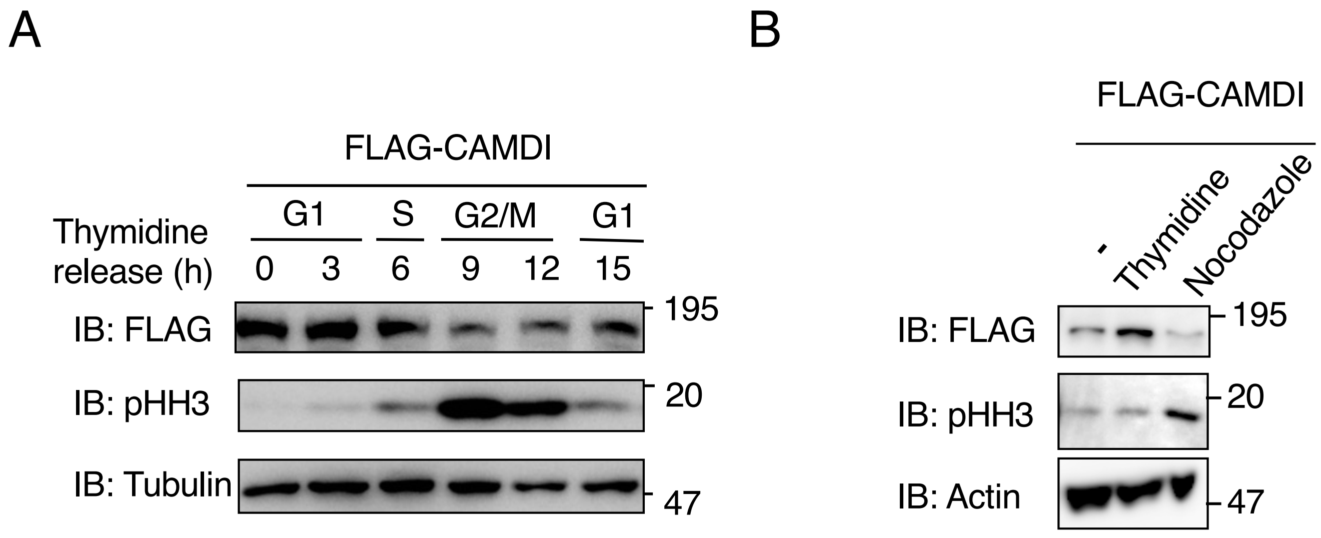

Figure S5. CAMDI degradation occurs in the G_2/M phase

(A) CAMDI protein level decreased from G_2 to mitosis. HeLa cells were transfected with FLAG-CAMDI, synchronized at the G_1/S phase by 2.5 mM double-thymidine block and then released into the cell cycle. Samples of each time point were analyzed by immunoblot assay using the indicated antibody.

(B) CAMDI protein level stabilized at the G_1 phase and destabilized at the G_2/M phase. HeLa cells were transfected with FLAG-CAMDI and synchronized at the G_1/S phase by double-thymidine block or at the G_2/M phase by treatment with 100 ng/ml nocodazole for 18 h. Cells were harvested and analyzed by immunoblotting using the indicated antibodies.

Figure S6

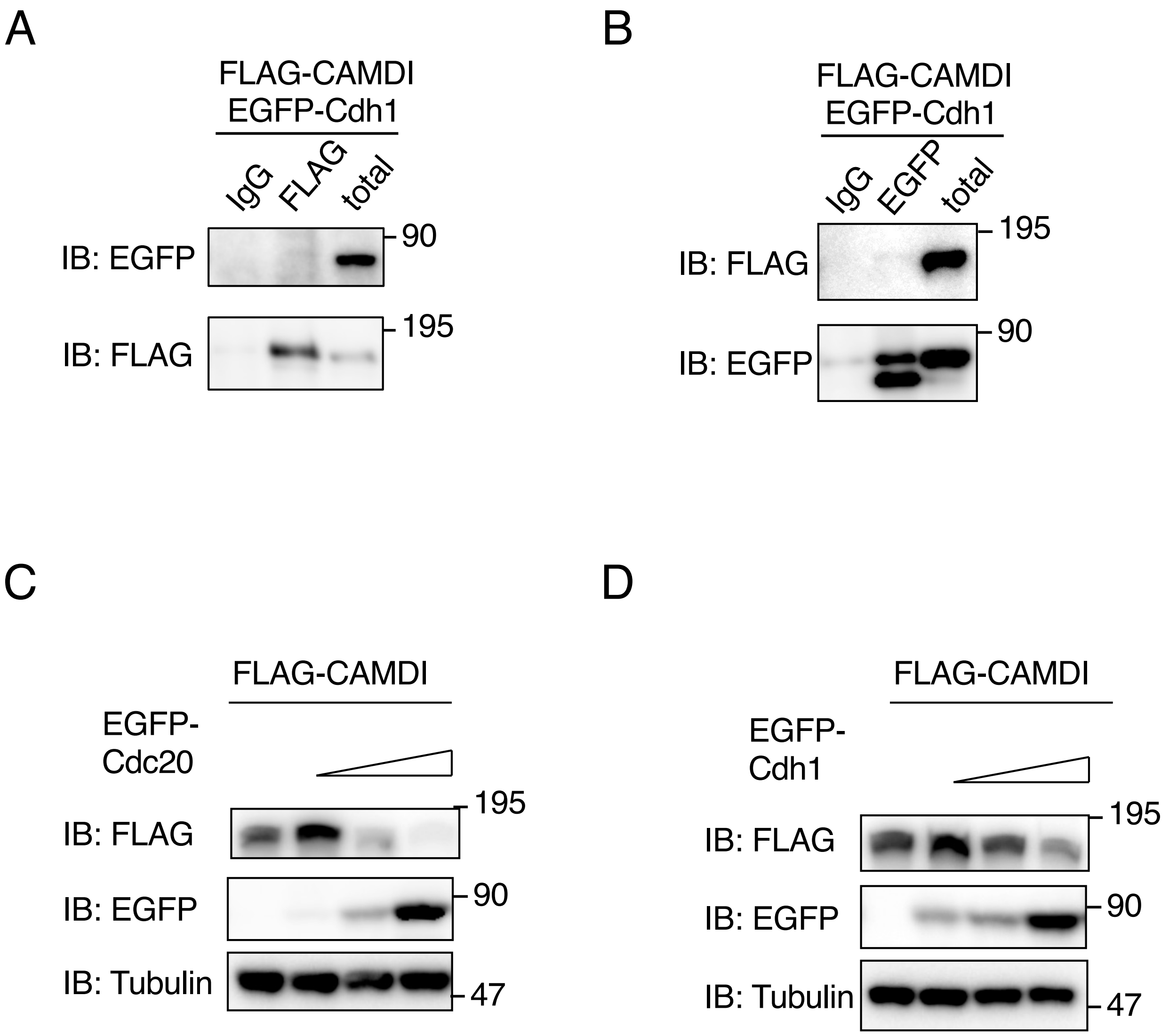

Figure S6. CAMDI degradation occurs in a Cdh1-independent manner

(A, B) CAMDI failed to interact with Cdh1. HeLa cells were transfected with FLAG-CAMDI and EGFP-Cdh1 plasmids. Cell lysates were subjected to immunoblot assay with the indicated antibodies. n=3 independent experiments.

(C, D) Cdh1 degraded CAMDI with weaker affinity than Cdc20. HeLa cells were transfected with FLAG-CAMDI and varying quantities of EGFP-Cdc20 or EGFP-Cdh1 plasmids. Cell lysates were subjected to immunoprecipitation assay with anti-FLAG antibody (A) or anti-EGFP antibody (B) and immunoblot assay. n=3 independent experiments.

Figure S7

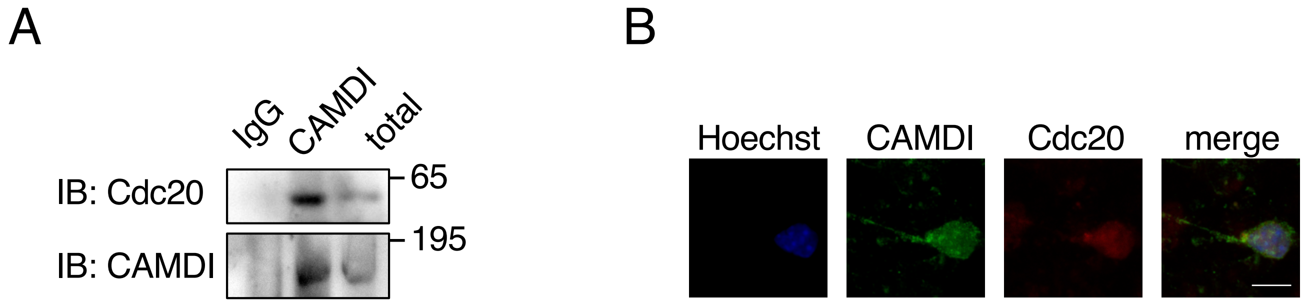

Figure S7. CAMDI interacts and colocalize with Cdc20 endogenously

(A) CAMDI interacted with Cdc20 in cortical neurons. E14.5 brain lysate was subjected to immunoprecipitation and immunoblot assays. n=3 independent experiments.

(B) CAMDI colocalized with Cdc20 in E14.5 primary cortical neurons at DIV2. Cells were subjected to immunocytochemical analysis using anti-CAMDI and anti-Cdc20 antibodies. Counterstaining with Hoechst was performed to visualize the nucleus. Scale bar 10 μm.

Figure S8

A

123		660	
Homo sapiens	RRTELLRLT	*Homo sapiens*	EREELSLLR
Equus caballus	KRRQLGRLT	*Equus caballus*	EREELGRLR
Canis lupus	RRRELSHLT	*Canis lupus*	EREELSHLR
Mus musculus	RRRELSHLT	*Mus musculus*	GREELSHLR
Consensus	.RxxLxxL/I/V/M.	Consensus	.RxxLxxL/I/V/M.
Mutant	.**A**xx**A**xxL	Mutant	.**A**xx**A**xxL

B

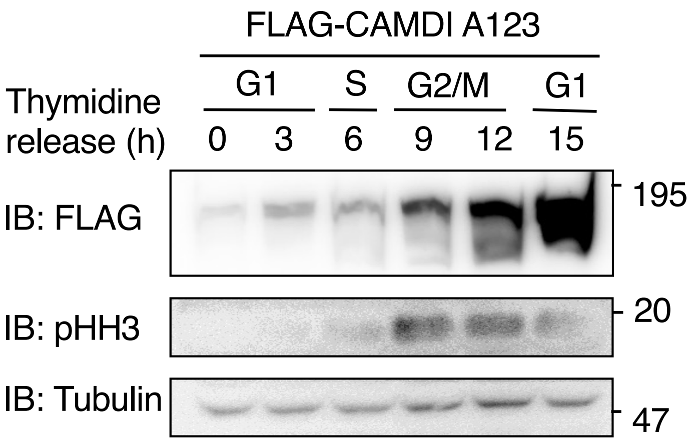

Figure S8. D-box consensus sequence of CAMDI

(A) Multiple alignment of amino acid sequence containing the consensus D-box sites within CAMDI. Here, 123 and 660 are positions of the first consensus arginine (R) residue.

(B) CAMDI A123 mutant protein level failed to decrease from G_2 to mitosis. HeLa cells were transfected with FLAG-CAMDI, synchronized at the G_1/S phase by 2.5 mM double-thymidine block and released into the cell cycle. Samples of each time point were analyzed by immunoblot assay using the indicated antibody.

Figure S9

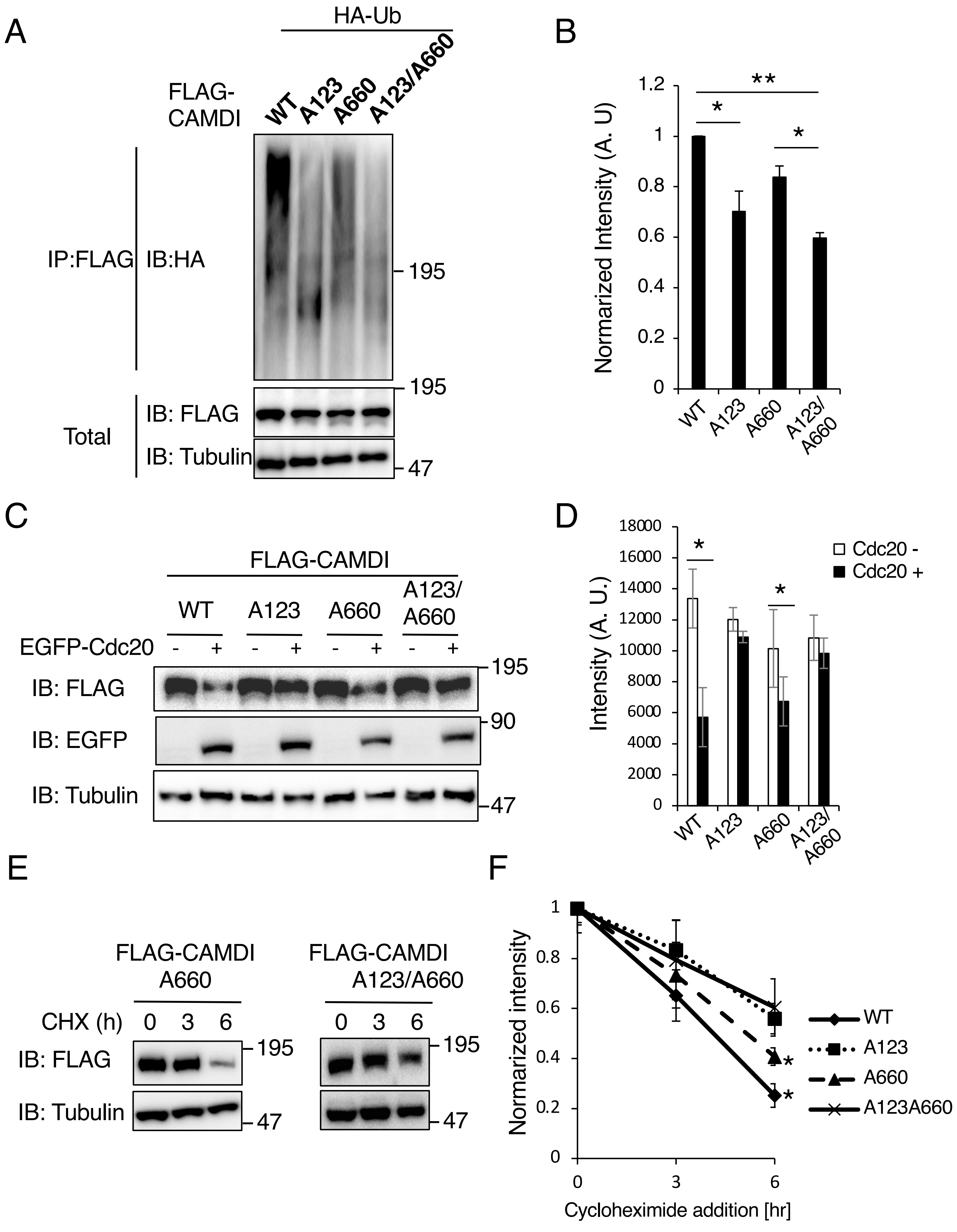
A
HA-Ub
FLAG-CAMDI
WT
A123
A660
A123/A660
IP:FLAG
IB:HA
195
Total
IB: FLAG
IB: Tubulin
47
B
Normarized Intensity (A. U)
1.2
1
0.8
0.6
0.4
0.2
0
WT
A123
A660
A123/A660
*
**
C
FLAG-CAMDI
WT
A123
A660
A123/A660
EGFP-Cdc20
- + - + - + - +
IB: FLAG
IB: EGFP
IB: Tubulin
195
90
47
D
Intensity (A. U.)
18000
16000
14000
12000
10000
8000
6000
4000
2000
0
Cdc20 -
Cdc20 +
E
FLAG-CAMDI A660
FLAG-CAMDI A123/A660
CHX (h)
0 3 6
IB: FLAG
IB: Tubulin
F
Normarized intensity
Cycloheximide addition [hr]
0
3
6
WT
A123
A660
A123A660

Figure S9. CAMDI A123 is a stable mutant to Cdc20-APC/C

(A) CAMDI was ubiquitinated in a D-box-dependent manner. HeLa cells were transfected with FLAG-CAMDI WT (full length), A123 (D-box at 123 a.a.), A660 (D-box at 660 a.a.) or A123/A660 double mutant and HA-Ub plasmids. Cell lysates were immunoprecipitated with anti-FLAG antibody and subjected to immunoblot assay using the indicated antibody.

(B) Quantification of band intensity of (A). n = 3 independent experiments. *, $p<0.05$; **, $p<0.01$, two-way ANOVA followed by Scheffe's *post hoc* test. Data are presented as mean ± SD.

(C) CAMDI WT and A660, but not A123 and A123/A660 mutants, were degraded by Cdc20 co-expression.

(D) Quantification of band intensity of (C). n = 3 independent experiments. *, $p<0.05$, two-way ANOVA followed by Scheffe's post hoc test. Data are presented as mean ± SD.

(E) Cycloheximide (CHX)-chase assay indicated the rapid degradation of CAMDI A660, but not A123/A660 D-box mutants. HeLa cells transfected with indicated plasmids were treated with CHX (10 μg/ml) for the indicated times, and each lysate was subjected to immunoblotting assay.

(F) The relative protein levels of FLAG-CAMDI in (E) and Figure 5C were quantified by densitometry. n=3 independent experiments. *, $p<0.05$, two-way ANOVA followed by Scheffe's post hoc test. Data are presented as mean ± SD.

Figure S10

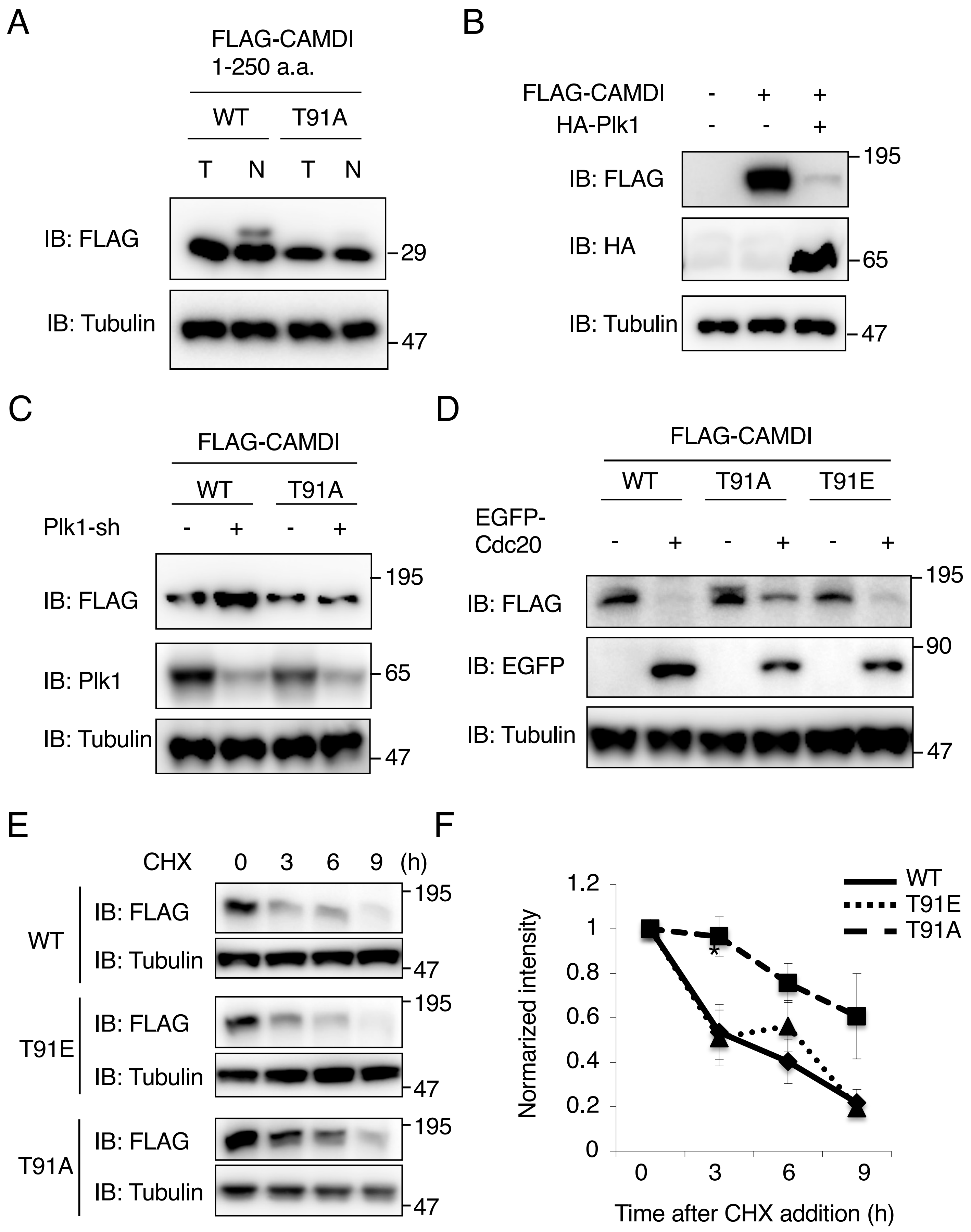
A
FLAG-CAMDI
1-250 a.a.
WT
T91A
T N T N
IB: FLAG
IB: Tubulin
29
47
B
FLAG-CAMDI - + +
HA-Plk1 - - +
IB: FLAG
IB: HA
IB: Tubulin
195
65
47
C
FLAG-CAMDI
WT
T91A
Plk1-sh - + - +
IB: FLAG
IB: Plk1
IB: Tubulin
195
65
47
D
FLAG-CAMDI
WT
T91A
T91E
EGFP-
Cdc20
- + - + - +
IB: FLAG
IB: EGFP
IB: Tubulin
195
90
47
E
CHX 0 3 6 9 (h)
WT
T91E
T91A
IB: FLAG
IB: Tubulin
195
47
F
WT
T91E
T91A
Normarized intensity
Time after CHX addition (h)
1.2
1
0.8
0.6
0.4
0.2
0
0
3
6
9
*

Figure S10. CAMDI is phosphorylated and destabilized by Plk1

(A) N' of CAMDI (1–250 a.a.) was detected as a band shift at the G_2/M phase. HeLa cells were transfected with FLAG-CAMDI (1–250 a.a.) and synchronized at the G_1 (thymidine: T) or G_2/M (nocodazole: N) phase. Cell lysates were subjected to immunoblot analysis.

(B) CAMDI was destabilized upon co-transfection of Plk1. HeLa cells were transfected with CAMDI (WT) with/without HA-Plk1 and cell lysates were analyzed by immunoblot assay.

(C) CAMDI was stabilized by Plk1 knockdown. HeLa cells were transfected with FLAG-CAMDI WT or non-phospho-mimetic CAMDI T91A with or without Plk1-sh and cell lysates were analyzed by immunoblot assay.

(D) Non-phospho-mimetic CAMDI (T91A) was resistant to degradation by Cdc20. HeLa cells were transfected with FLAG-CAMDI (WT), FLAG-CAMDI (T91A) or FLAG-CAMDI (T91E), with the replacement of threonine by glutamate at position 91 for phospho-mimetic properties, and with/without EGFP-Cdc20. Cell lysates were subjected to immunoblot analysis.

(E) Cycloheximide (CHX)-chase assay indicated the resistance to degradation via T91 phosphorylation. HeLa cells transfected with indicated vectors were treated with CHX (10 μg/ml) for the indicated times, and each lysate was subjected to immunoblotting assay.

(F) The relative protein levels of FLAG-CAMDI in (E) were quantified by densitometry. n=3 independent experiments. *, $p<0.05$, two-way ANOVA followed by Scheffe's *post hoc* test. Data are presented as mean ± SD.

Figure S11

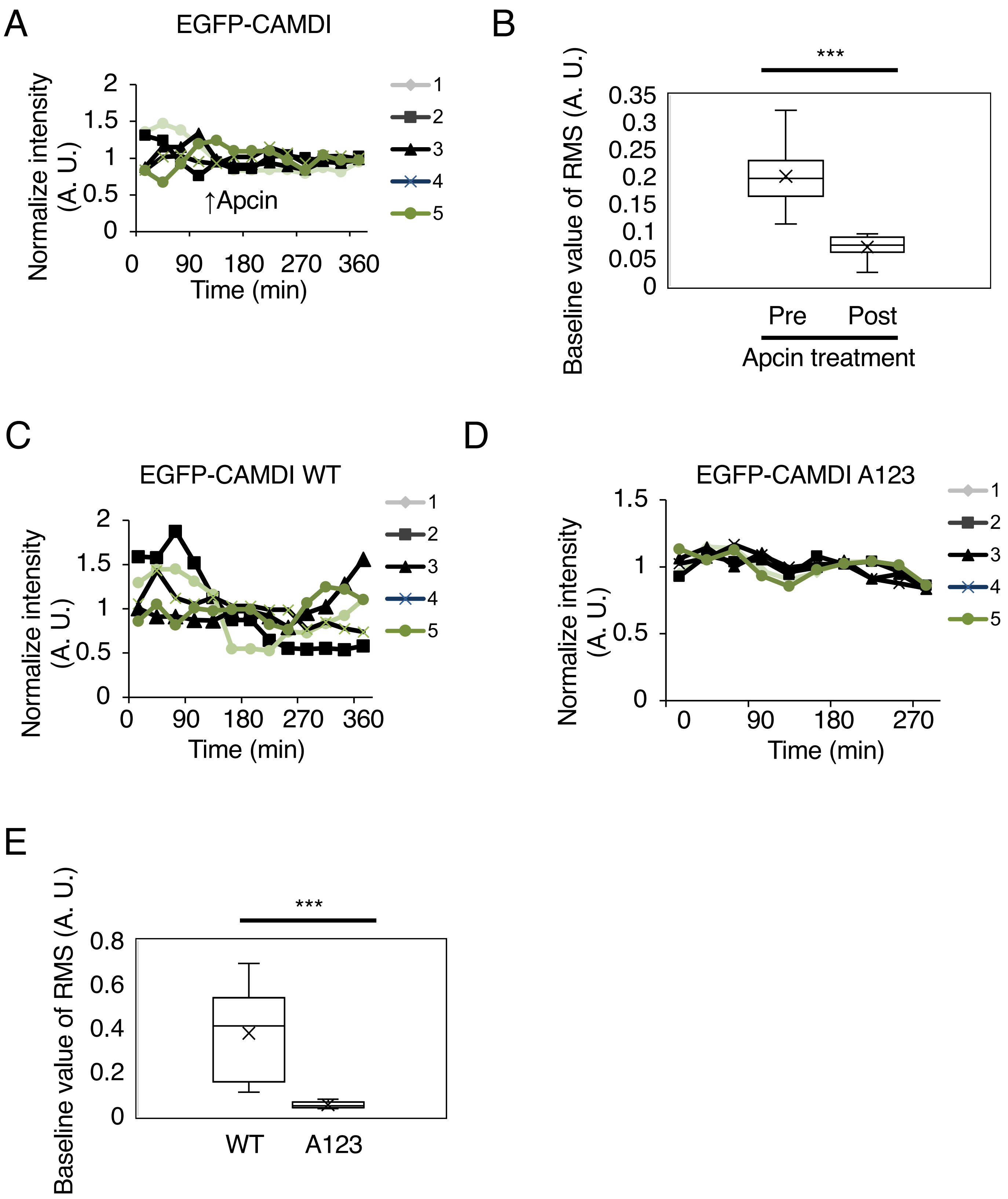

A
EGFP-CAMDI
Normalize intensity (A. U.)
2
1.5
1
0.5
0
↑Apcin
0
90
180
270
360
Time (min)
1
2
3
4
5
B
***
Baseline value of RMS (A. U.)
0.35
0.3
0.25
0.2
0.15
0.1
0.05
0
Pre
Post
Apcin treatment
C
EGFP-CAMDI WT
Normalize intensity (A. U.)
2
1.5
1
0.5
0
0
90
180
270
360
Time (min)
1
2
3
4
5
D
EGFP-CAMDI A123
Normalize intensity (A. U.)
1.5
1
0.5
0
0
90
180
270
Time (min)
1
2
3
4
5
E
***
Baseline value of RMS (A. U.)
0.8
0.6
0.4
0.2
0
WT
A123

Figure S11. Normalized intensity and Box-and-whisker plot analysis of electroporated migrating neuron

(A) Normalized intensity of EGFP-CAMDI with Apcin treatment related to Figure 6D.

(B) Box-and-whisker plot analysis of Baseline value of the RMS (the root mean square). N=10 cells. ***, $p<0.001$.

(C, D) Normalized intensity of EGFP-CAMDI WT (C) and EGFP-CAMDI A123 (D) with related to Figure 7C and D.

(E) Box-and-whisker plot analysis of Baseline value of the RMS (the root mean square). N=10 cells. ***, $p<0.001$.
